# Coded Caching for Broadcast Networks with User Cooperation †

**DOI:** 10.3390/e24081034

**Published:** 2022-07-27

**Authors:** Zhenhao Huang, Jiahui Chen, Xiaowen You, Shuai Ma, Youlong Wu

**Affiliations:** 1 School of Information Science and Technology, ShanghaiTech University, No. 393 Huaxia Middle Road, Pudong, Shanghai 201210, China; huangzhh@shanghaitech.edu.cn (Z.H.); chenjh1@shanghaitech.edu.cn (J.C.); youxw@shanghaitech.edu.cn (X.Y.); 2 Information Processing and Communications Laboratory, Telecom Paris, IP Paris, 91120 Palaiseau, France; ma@telecom-paris.fr

**Keywords:** coded cache, cooperation, device-to-device, transmission delay

## Abstract

Caching technique is a promising approach to reduce the heavy traffic load and improve user latency experience for the Internet of Things (IoT). In this paper, by exploiting edge cache resources and communication opportunities in device-to-device (D2D) networks and broadcast networks, two novel coded caching schemes are proposed that greatly reduce transmission latency for the centralized and decentralized caching settings, respectively. In addition to the multicast gain, both schemes obtain an additional *cooperation gain* offered by user cooperation and an additional *parallel gain* offered by the parallel transmission among the server and users. With a newly established lower bound on the transmission delay, we prove that the centralized coded caching scheme is *order-optimal*, i.e., achieving a constant multiplicative gap within the minimum transmission delay. The decentralized coded caching scheme is also order-optimal if each user’s cache size is larger than a threshold which approaches zero as the total number of users tends to infinity. Moreover, theoretical analysis shows that to reduce the transmission delay, the number of users sending signals simultaneously should be appropriately chosen according to the user’s cache size, and always letting more users send information in parallel could cause high transmission delay.

## 1. Introduction

With the rapid development of Internet of Things (IoT) technologies, IoT data traffic, such as live streaming and on-demand video streaming, has grown dramatically over the past few years. To reduce the traffic load and improve the user latency experience, the caching technique has been viewed as a promising approach that shifts the network traffic to low congestion periods. In the seminal paper [1], Maddah-Ali and Niesen proposed a coded caching scheme based on centralized file placement and coded multicast delivery that achieves a significantly larger global multicast gain compared to the conventional uncoded caching scheme.

The coded caching scheme has attracted wide and significant interest. The coded caching scheme was extended to a setup with decentralized file placement, where no coordination is required for the file placement [2]. For the cache-aided broadcast network, ref. [3] showed that the rate–memory tradeoff of the above caching system is within a factor of 2.00884. For the setting with uncoded file placement where each user stores uncoded content from the library, refs. [4,5] proved that Maddah-Ali and Niesen’s scheme is optimal. In [6], both the placement and delivery phases of coded caching are depicted using a placement delivery array (PDA), and an upper bound for all possible regular PDAs was established. In [7], the authors studied a cached-aided network with heterogeneous setting where the user cache memories are unequal. More asymmetric network settings have been discussed, such as coded caching with heterogeneous user profiles [8], with distinct sizes of files [9], with asymmetric cache sizes [10,11,12] and with distinct link qualities [13]. The settings with varying file popularities have been discussed in [14,15,16]. Coded caching that jointly considers various heterogeneous aspects was studied in [17]. Other works on coded caching include, e.g., cache-aided noiseless multi-server network [18], cache-aided wireless/noisy broadcast networks [19,20,21,22], cache-aided relay networks [23,24,25], cache-aided interference management [26,27], coded caching with random demands [28], caching in combination networks [29], coded caching under secrecy constraints [30], coded caching with reduced subpacketization [31,32], the coded caching problem where each user requests multiple files [33], and a cache-aided broadcast network for correlated content [34], etc.

A different line of work is to study the cached-aided networks without the presence of a server, e.g., the device-to-device (D2D) cache-aided network. In [35], the authors investigated coded caching for wireless D2D network [35], where users locate in a fixed mesh topology wireless D2D network. A D2D system with selfish users who do not participate in delivering the missing subfiles to all users was studied in [36]. Wang et al. applied the PDA to characterize cache-aided D2D wireless networks in [37]. In [38], the authors studied the spatial D2D networks in which the user locations are modeled by a Poisson point process. For heterogeneous cache-aided D2D networks where users are equipped with cache memories of distinct sizes, ref. [39] minimized the delivery load by optimizing over the partition during the placement phase and the size and structure of D2D during the delivery phase. A highly dense wireless network with device mobility was investigated in [40].

In fact, combining the cache-aided broadcast network with the cache-aided D2D network can potentially reduce the transmission latency. This hybrid network is common in many practical distributed systems such as cloud network [41], where a central cloud server broadcasts messages to multiple users through the cellular network, and meanwhile users communicate with each other through a fiber local area network (LAN). A potential scenario is that users in a moderately dense area, such as a university, want to download files, such as movies, from a data library, such as a video service provider. It should be noted that the user demands are highly redundant, and the files need not only be stored by a central server but also partially cached by other users. Someone can attain the desired content through both communicating with the central server and other users such that the communication and storage resources can be used efficiently. Unfortunately, there is very little research investigating the coded caching problem for this hybrid network. In this paper, we consider such hybrid cache-aided network where a server consisting of $N\in Z^{+}$ files connects with $K\in Z^{+}$ users through a broadcast network, and meanwhile the users can exchange information via a D2D network. Unlike the settings of [35,38], in which each user can only communicate with its neighboring users via spatial multiplexing, we consider the D2D network as either an error-free shared link or a flexible routing network [18]. In particular, for the case of the shared link, all users exchange information via a shared link. In the flexible routing network, there exists a routing strategy adaptively partitioning all users into multiple groups, in each of which one user sends data packets error-free to the remaining users in the corresponding group. Let α∈Z be the number of groups who send signals at the same time, then the following fundamental questions arise for this hybrid cache-aided network:

- *How does α affect the system performance?*
- *What is the (approximately) optimal value of α to minimize the transmission latency?*
- *How can communication loads be allocated between the server and users to achieve the minimum transmission latency?*

In this paper, we try to address these questions, and our main contributions are summarized as follows:

- We propose novel coded caching schemes for this hybrid network under centralized and decentralized data placement. Both schemes efficiently exploit communication opportunities in D2D and broadcast networks, and appropriately allocate communication loads between the server and users. In addition to multicast gain, our schemes achieve much smaller transmission latency than both that of Maddah-Ali and Niesen’s scheme for a broadcast network [1,2] and the D2D coded caching scheme [35]. We characterize a *cooperation gain* and a *parallel gain* achieved by our schemes, where the cooperation gain is obtained through cooperation among users in the D2D network, and the parallel gain is obtained through the parallel transmission between the server and users.
- We prove that the centralized scheme is order-optimal, i.e., achieving the optimal transmission delay within a constant multiplicative gap in all regimes. Moreover, the decentralized scheme is also optimal when the cache size of each user *M* is larger than the threshold $N(1-\sqrt[{K-1}]{1/\left(K+1\right)})$ that is approaching zero as K→∞.
- For the centralized data placement case, theoretical analysis shows that α should decrease with the increase of the user caching size. In particular, when each user’s caching size is sufficiently large, only one user should be allowed to send information, indicating that the D2D network can be just a simple shared link connecting all users. For the decentralized data placement case, α should be dynamically changing according to the sizes of subfiles created in the placement phase. In other words, always letting more users parallelly send information can cause a high transmission delay.

Please note that the decentralized scenario is much more complicated than the centralized scenario, since each subfile can be stored by s=1,2,…,K users, leading to a dynamic file-splitting and communication strategy in the D2D network. Our schemes, in particular the decentralized coded caching scheme, differ greatly with the D2D coded caching scheme in [35]. Specifically, ref. [35] considered a fixed network topology where each user connects with a fixed set of users, and the total user cache sizes must be large enough to store all files in the library. However, in our schemes, the user group partition is dynamically changing, and each user can communicate with any set of users via network routing. Moreover, our model has the server share communication loads with the users, resulting in an allocation problem on communication loads between the broadcast network and D2D network. Finally, our schemes achieve a tradeoff between the cooperation gain, parallel gain and multicast gain, while the schemes in [1,2,35] only achieve the multicast gain.

The remainder of this paper is as follows. Section 2 presents the system model, and defines the main problem studied in this paper. We summarize the obtained main results in Section 3. Following that is a detailed description of the centralized coded caching scheme with user cooperation in Section 4. Section 5 extends the techniques we developed for the centralized caching problem to the setting of decentralized random caching. Section 6 concludes this paper.

## 2. System Model and Problem Definition

**Figure 1:**
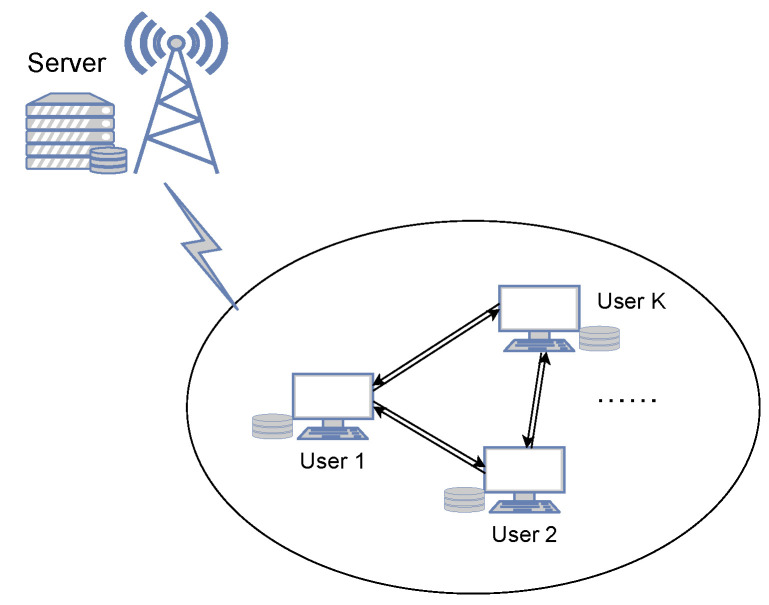
Caching system considered in this paper. A server connects with *K* cache-enabled users and the users can cooperate through a flexible network.

Consider a cache-aided network consisting of a single server and *K* users as depicted in Figure 1. The server has a library of *N* independent files $W_{1},\ldots ,W_{N}$. Each file $W_{n}$, n=1,…,N, is uniformly distributed over

$$\left[2^{F}\right]≜\left\{1,2,\ldots ,2^{F}\right\},\;$$

for some positive integer *F*. The server connects with *K* users through a noisy-free shared link but rate-limited to a network speed of $C_{1}$ bits per second (bits/s). Each user k∈[K] is equipped with a cache memory of size MF bits, for some M∈[0,N], and can communicate with each other via a D2D network.

We mainly focus on two types of D2D networks: a shared link as in [1,2] and a flexible routing network introduced in [18]. In the case of a shared link, all users connect with each other through a shared error-free link but rate-limited to $C_{2}$ bits/s. In the flexible routing network, *K* users can arbitrarily form multiple groups via network routing, in each of which at most one user can send error-free data packets at a network speed $C_{2}$ bits/s to the remaining users within the group. To unify these two types of D2D networks, we introduce an integer $\alpha_{\max}\in \left\{1,\left\lfloor \frac{K}{2}\right\rfloor \right\}$, which denotes the maximum number of groups allowed to send data parallelly in the D2D network. For example, when $\alpha_{\max}=1$, the D2D network degenerates into a shared link, and when $\alpha_{\max}=\left\lfloor \frac{K}{2}\right\rfloor$, it turns to be the flexible network.

The system works in two phases: a placement phase and a delivery phase. In the placement phase, all users will access the entire library $W_{1},\ldots ,W_{N}$ and fill the content to their caching memories. More specifically, each user *k*, for k∈[K], maps $W_{1},\ldots ,W_{N}$ to its cache content:

(1) $$\begin{array}{c} Z_{k}≜\phi_{k}\left(W_{1},\ldots ,W_{N}\right), \end{array}$$

for some caching function

(2) $$\begin{array}{c} \phi_{k}:{\left[2^{F}\right]}^{N}\to \left[\left\lfloor 2^{MF}\right\rfloor \right]. \end{array}$$

In the delivery phase, each user requests one of the *N* files from the library. We denote the demand of user *k* as $d_{k}\in \left[N\right]$, and its desired file as $W_{d_{k}}$. Let $d≜(d_{1},\ldots ,d_{K})$ denotes the request vector. In this paper, we investigate the worst request case where each user makes a unique request.

Once the request vector d is informed to the server and all users, the server produces the symbol

(3) $$\begin{array}{c} X≜f_{d}\left(W_{1},\ldots ,W_{N}\right), \end{array}$$

and broadcasts it to all users through the broadcast network. Meanwhile, user k∈{1,…,K} produces the symbol (Each user *k* can produce $X_{k}$ as a function of $Z_{k}$ and the received signals sent by the server, but because all users can access to the server’s signal due to the fact that the server broadcasts its signals to the network, it is equivalent to generating $X_{k}$ as a function $Z_{k}$).

(4) $$\begin{array}{c} X_{k}≜f_{k,d}\left(Z_{k}\right), \end{array}$$

and sends it to a set of intended users $D_{k}\subseteq \left[K\right]$ through the D2D network. Here, $D_{k}$ represents the set of destination users served by node *k*, $f_{d}$ and $f_{k,d}$ are some encoding functions

(5) $$\begin{array}{ccc} & & f_{d}:{\left[2^{F}\right]}^{N}\to \left[\left\lfloor 2^{R_{1}F}\right\rfloor \right],\;f_{k,d}:\left[\left\lfloor 2^{MF}\right\rfloor \right]\to \left[\left\lfloor 2^{R_{2}F}\right\rfloor \right],\; \end{array}$$

where $R_{1}$ and $R_{2}$ denote the *transmission rate* sent by the server in the broadcast network and by each user in the D2D network, respectively. Here we focus on the symmetric case where all users have the same transmission rate. Due to the constraint of $\alpha_{\max}$, at most $\alpha_{\max}$ users can send signals parallelly in each channel use. The set of $\alpha_{\max}$ users who send signals in parallel could be adaptively changed in the delivery phase.

At the end of the delivery phase, due to the error-free transmission in the broadcast and D2D networks, user *k* observes symbols sent to them, i.e., $\left(X_{j}:j\in \left[K\right],k\in D_{j}\right)$, and decodes its desired message as ${\hat{W}}_{d_{k}}=\psi_{k,d}\left(X,\left(X_{j}:j\in \left[K\right],k\in D_{j}\right),Z_{k}\right),$ where $\psi_{k,d}$ is a decoding function.

We define the worst-case probability of error as

(6) $$\begin{array}{c} P_{e}≜\max_{d\in F^{n}}\max_{k\in [K]}\mathrm{Pr}\left({\hat{W}}_{d_{k}}\neq W_{d_{k}}\right). \end{array}$$

A coded caching scheme $\left(M,R_{1},R_{2}\right)$ consists of caching functions $\left\{\phi_{k}\right\}$, encoding functions $\left\{f_{d},f_{k,d}\right\}$ and decoding functions $\left\{\psi_{k,d}\right\}$. We say that the rate region $\left(M,R_{1},R_{2}\right)$ is *achievable* if for every ϵ>0 and every large enough file size *F*, there exists a coded caching scheme such that $P_{e}$ is less than ϵ.

Since the server and the users send signals in parallel, the total transmission delay, denoted by *T*, can be defined as

(7) $$\begin{array}{c} T≜\max\{\frac{R_{1}F}{C_{1}},\frac{R_{2}F}{C_{2}}\}. \end{array}$$

The *optimal* transmission delay is $T^{*}≜\inf\left\{T:T\;\mathrm{is}\;\mathrm{achievable}\right\}$. For simplicity, we assume that $C_{1}=C_{2}=F$, and then from (7) we have

(8) $$\begin{array}{c} T=\max\{R_{1},R_{2}\}. \end{array}$$

When $C_{1}\neq C_{2}$, e.g., $C_{1}:C_{2}=1/k$, one small adjustment allowing our scheme to continue to work is multiplying λ by 1/(k(1−λ)+λ), where λ is a devisable parameter introduced later.

Our goal is to design a coded caching scheme to minimize the transmission delay. Finally, in this paper we assume K≤N and M≤N. Extending the results to other scenarios is straightforward, as mentioned in [1].

## 3. Main Results

We first establish a general lower bound on the transmission delay for the system model described in Section 2, then present two upper bounds of the optimal transmission delay achieved by our centralized and decentralized coded caching schemes, respectively. Finally, we present the optimality results of these two schemes.

**Theorem** **1** (Lower Bound)**.** *For memory size 0≤M≤N, the optimal transmission delay is lower bounded by*

(9) $$\begin{array}{cc} T^{*}\geq \max & \left\{\frac{1}{2}\left(1-\frac{M}{N}\right),\max_{s\in [K]}\left(s-\frac{KM}{\left\lfloor N/s\right\rfloor }\right),\max_{s\in [K]}\left(s-\frac{sM}{\left\lfloor N/s\right\rfloor }\right)\frac{1}{1+\alpha_{\max}}\right\}. \end{array}$$

**Proof.**  See the proof in Appendix A. □

### 3.1. Centralized Coded Caching

In the following theorem, we present an upper bound on the transmission delay for the centralized caching setup.

**Theorem** **2** (Upper Bound for the Centralized Scenario)**.** Let $t≜KM/N\in Z^{+}$, and $\alpha \in Z^{+}$. For memory size $M\in \{0,\frac{N}{K},\frac{2N}{K},\ldots ,N\}$, the optimal transmission delay $T^{*}$ is upper bounded by $T^{*}\leq T_{\mathrm{central}}$, where

(10) $$\begin{array}{c} T_{\mathrm{central}}≜\;\;\min_{\alpha \leq \alpha_{\max}}\;\;K\left(1\;-\;\frac{M}{N}\right)\frac{1}{1\;+\;t\;+\;\alpha \min\{\left\lfloor \frac{K}{\alpha }\right\rfloor \;-\;1,t\}}.\; \end{array}$$

*For general 0≤M≤N, the lower convex envelope of these points is achievable.*

**Proof.**  See scheme in Section 4. □

The following simple example shows that the proposed upper bound can greatly reduce the transmission delay.

**Example** **1.** *Consider a network described in Section 2 with KM/N=K−1. The coded caching scheme without D2D communication [1] has the server multicast an XOR message useful for all K users, achieving the transmission delay $K\left(1-\frac{M}{N}\right)\frac{1}{1+t}=\frac{1}{K}$. The D2D coded caching scheme [35] achieves the transmission delay $\frac{N}{M}\left(1-\frac{M}{N}\right)=\frac{1}{K-1}$. The achievable transmission delay in Theorem 2 equals $\frac{1}{2K-1}$ by letting α=1, almost twice as short as the transmission delay of previous schemes if K is sufficiently large.*

From (10), we obtain that the optimal value of α, denoted by $\alpha^{*}$, equals 1 if t≥K−1 and to $\alpha_{\max}$ if $t\leq \lfloor \frac{K}{\alpha_{\max}}\rfloor \;-\;1$. When ignoring all integer constraints, we obtain $\alpha^{*}=\frac{K}{t+1}$. We rewrite this choice as follows:

(11) $$\alpha^{*}=\;\left\{\begin{array}{ccc} \; & 1, & t\geq K-1,\\ \; & K/\left(t+1\right), & \lfloor K/\alpha_{\max}\rfloor \;-\;1\;<\;t\;<\;K\;-\;1,\\ \; & \alpha_{\max}, & t\leq \lfloor K/\alpha_{\max}\rfloor \;-\;1. \end{array}\right.$$

**Remark** **1.** From (11), we observe that when M is small such that $t\leq \lfloor K/\alpha_{\max}\rfloor \;-\;1$, we have $\alpha^{*}=\alpha_{\max}$. As M is increasing, $\alpha^{*}$ becomes K/(t+1), smaller than $\alpha_{\max}$. When M is sufficiently large such that M≥(K−1)N/K, only one user should be allowed to send information, i.e., $\alpha^{*}=1$. This indicates that letting more users parallelly send information could be harmful. The main reason for this phenomenon is the existence of a tradeoff between the multicast gain, cooperation gain* and* parallel gain, *which will be introduced below in this section*.

Comparing $T_{\mathrm{central}}$ with the transmission delay achieved by Maddah-Ali and Niesen’s scheme for the broadcast network [1], i.e., $K\left(1-\frac{M}{N}\right)\frac{1}{1+t}$, $T_{\mathrm{central}}$ consists of an additional factor

(12) $$\begin{array}{c} G_{\text{central,c}}≜\frac{1}{1+\frac{\alpha }{1+t}\min\left\{\left\lfloor \frac{K}{\alpha }\right\rfloor \;-\;1,t\right\}}, \end{array}$$

referred to as *centralized cooperation gain*, as it arises from user cooperation. Comparing $T_{\mathrm{central}}$ with the transmission delay achieved by the D2D coded caching scheme [35], i.e., $\frac{N}{M}\left(1-\frac{M}{N}\right)$, $T_{\mathrm{central}}$ consists of an additional factor

(13) $$\begin{array}{c} G_{\text{central,p}}≜\frac{1}{1+\frac{1}{t}+\frac{\alpha }{t}\min\left\{\left\lfloor \frac{K}{\alpha }\right\rfloor \;-\;1,t\right\}}, \end{array}$$

referred to as *centralized parallel gain*, as it arises from parallel transmission among the server and users. Both gains depend on *K*, M/N and $\alpha_{\max}$.

Substituting the optimal $\alpha^{*}$ into (12), we have

(14) $$G_{\text{central,c}}=\;\left\{\begin{array}{ccc} \; & \frac{1+t}{K+t}, & t\geq K-1,\\ \; & \frac{1+t}{K-\frac{K}{t+1}\;+\;t}, & \lfloor \frac{K}{\alpha_{\max}}\rfloor \;-\;1\;<\;t\;<\;K\;-\;1,\\ \; & \frac{1+t}{\alpha_{\max}t+t+1}, & t\leq \lfloor \frac{K}{\alpha_{\max}}\rfloor \;-\;1. \end{array}\right.$$

When fixing $\left(K,N,\alpha_{\max}\right)$, $G_{\text{central,c}}$ in general is not a monotonic function of *M*. More specifically, when *M* is small enough such that $t<\lfloor \frac{K}{\alpha_{\max}}\rfloor \;-\;1$, the function $G_{\text{central,c}}$ is monotonically decreasing, indicating that the improvement caused by introducing D2D communication. This is mainly because relatively larger *M* allows users to share more common data with each other, providing more opportunities on user cooperation. However, when *M* grows larger such that $t\geq \lfloor \frac{K}{\alpha_{\max}}\rfloor \;-\;1$, the local and global caching gains become dominant, and less improvement can be obtained from user cooperation, turning $G_{\text{central,c}}$ to a monotonic increasing function of *M*,

Similarly, substituting the optimal $\alpha^{*}$ into (13), we obtain

(15) $$G_{\text{central,p}}=\;\left\{\begin{array}{ccc} \; & \frac{t}{K+t}, & t\geq K-1,\\ \; & \frac{t}{\frac{t\cdot K}{t+1}+t+1}, & \lfloor \frac{K}{\alpha_{\max}}\rfloor \;-\;1\;<\;t\;<\;K\;-\;1,\\ \; & \frac{t}{\alpha_{\max}t+t+1}, & t\leq \lfloor \frac{K}{\alpha_{\max}}\rfloor \;-\;1. \end{array}\right.$$

Equation (15) shows that $G_{\text{central,p}}$ is monotonically increasing with *t*, mainly due to the fact that when *M* increases, more content can be sent through the D2D network without the help of the central server, decreasing the improvement from parallel transmission between the server and users.

**Figure 2:**
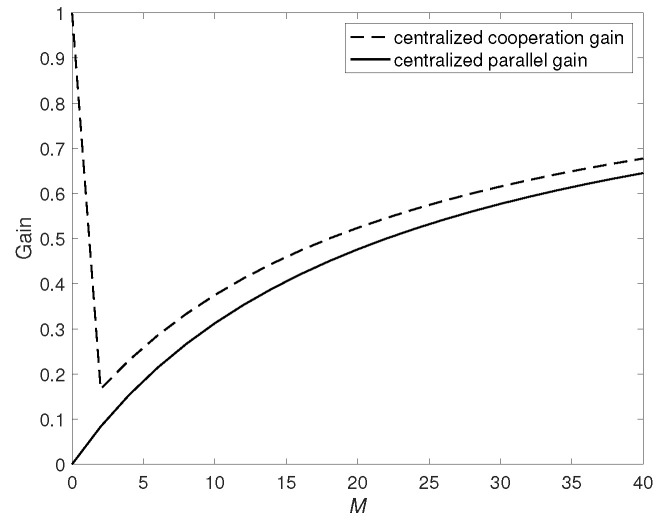
Centralized cooperation gain and parallel gain when N=40, K=20 and $\alpha_{\max}=5$.

The centralized cooperation gain (12) and parallel gain (13) are plotted in Figure 2 when N=40, K=20 and $\alpha_{\max}=5$.

**Remark** **2.** *Larger α could lead to better parallel and cooperation gain (more uses can concurrently multicast signals to other users), but will result in worse multicast gain (signals are multicast to fewer users in each group). The choice of α in (11) is in fact a tradeoff between the multicast gain, parallel gain and cooperation gain.*

The proposed scheme achieving the upper bound in Theorem 2 is order-optimal.

**Theorem** **3.** *For memory size 0≤M≤N,*

(16) $$\begin{array}{c} \frac{T_{\mathrm{central}}}{T^{*}}\leq 31. \end{array}$$

**Proof.**  See the proof in Appendix B. □

The exact gap of $T_{\mathrm{central}}/T^{*}$ could be much smaller. One could apply the method proposed in [3] to obtain a tighter lower bound and shrink the gap. In this paper, we only prove the order optimality of the proposed scheme, and leave the work of finding a smaller gap as the future work.

**Figure 3:**
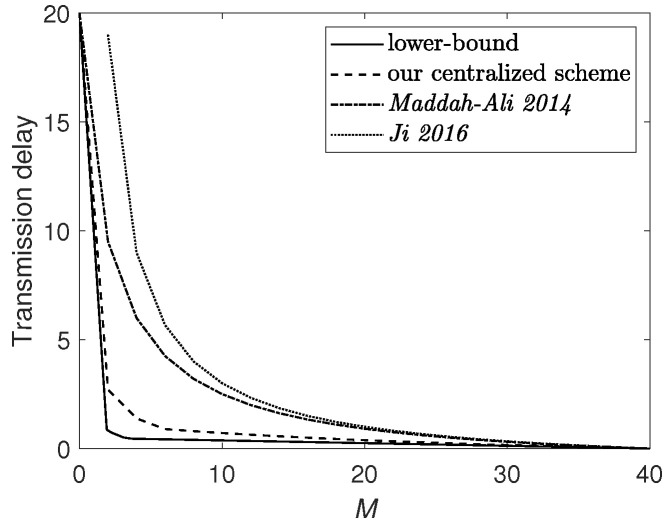
Transmission delay when N=40, K=20 and $\alpha_{\max}=5$. The upper bounds are achieved under the centralized caching scenario.

Figure 3 plots the lower bound (9) and upper bounds achieved by various schemes, including the proposed scheme, the scheme *Maddah-Ali 2014* in [1] which considers the broadcast network without D2D communication, and the scheme *Ji 2016* in [35], which considers the D2D network without server. It is obvious that our scheme outperforms the previous schemes and approaches closely to the lower bound.

### 3.2. Decentralized Coded Caching

We exploit the multicast gain from coded caching, D2D communication, and parallel transmission between the server and users, leading to the following upper bound.

**Theorem** **4** (Upper Bound for the Decentralized Scenario)**.** Define p≜M/N. For memory size 0≤M≤N, the optimal transmission delay $T^{*}$ is upper bounded by

(17) $$\begin{array}{c} T^{*}\leq T_{\mathrm{decentral}}≜\max\left\{R_{\emptyset },\frac{R_{s}R_{u}}{R_{s}+R_{u}-R_{\emptyset }}\right\}, \end{array}$$

*where*

(18) $$\begin{array}{cc} R_{\emptyset }≜ & K{\left(1-p\right)}^{K}, \end{array}$$

(19) $$\begin{array}{cc} R_{s}≜ & \frac{1-p}{p}\left(1-{\left(1-p\right)}^{K}\right), \end{array}$$

(20) Ru≜1αmax∑s=2⌈Kαmax⌉−1sKss−1ps−1(1−p)K−s+1+∑s=⌈Kαmax⌉KKK−1s−1f(K,s)ps−1(1−p)K−s+1,

*with*

(21) $$\begin{array}{c} f\left(K,s\right)≜\left\{\begin{array}{ccc} & \left\lfloor \frac{K}{s}\right\rfloor \left(s-1\right), & (K\;mod\;s)<2,\\ & K-1-\lfloor K/s\rfloor , & (K\;mod\;s)\geq 2. \end{array}\right. \end{array}$$

**Proof.**  Here, $R_{\emptyset }$ represents the transmission rate of sending contents that are not cached by any user, $R_{s}$ and $R_{u}$ represent the transmission rate sent by the server via the broadcast network, and the transmission rate sent by users via the D2D network, respectively. Equation (17) balances the communication loads assigned to the server and users. See more detailed proof in Section 5. □

The key idea of the scheme achieving (17) is to partition *K* users into $\left\lceil \frac{K}{s}\right\rceil$ groups for each communication round s∈[K−1], and let each group perform the D2D coded caching scheme [35] to exchange information. The main challenge is that that among all $\left\lceil \frac{K}{s}\right\rceil$ groups, there are $\left\lfloor \frac{K}{s}\right\rfloor$ groups of the same size *s*, and an *abnormal* group of size (Kmods) if (Kmods)≠0, leading to an asymmetric caching setup. One may use the scheme [35] for the groups of size *s*, for the group of size (Kmods)≥2, but how to exploit the caching resource and communication capability of all groups while balancing communication loads among the two types of groups to minimize the transmission delay remains elusive and needs to be carefully designed. Moreover, this challenge poses complexities both in establishing the upper bound and in optimality proof.

**Remark** **3.** *The upper bound in Theorem 4 is achieved by setting the number of users that exactly send signals in parallel as follows:*

(22) $$\alpha_{D}=\;\left\{\begin{array}{ccc} \; & \alpha_{\max},\; & \mathrm{case}\;1,\\ \; & \lfloor \frac{K}{s}\rfloor ,\; & \mathrm{case}\;2,\\ \; & \lceil \frac{K}{s}\rceil ,\; & \mathrm{case}\;3. \end{array}\right.$$

*If $\left\lceil \frac{K}{s}\right\rceil >\alpha_{\max}$, the number of users who send data in parallel is smaller than $\alpha_{\max}$, indicating that always letting more users parallelly send messages could cause higher transmission delay. For example, when K≥4, s=K−1 and $\alpha_{\max}=\left\lfloor \frac{K}{2}\right\rfloor$, we have $\alpha_{D}=1<\alpha_{\max}$.*

**Remark** **4.** *From the definitions of $T_{\mathrm{decentral}}$, $R_{s}$, $R_{u}$ and $R_{\emptyset }$, it is easy to obtain that $R_{\emptyset }\leq T_{\mathrm{decentral}}\leq R_{s}$,*

(23) $$\begin{array}{c} T_{\mathrm{decentral}}=\left\{\begin{array}{ccc} & \frac{R_{s}R_{u}}{R_{s}+R_{u}-R_{\emptyset }}, & R_{u}\geq R_{\emptyset },\\ & R_{\emptyset }, & R_{u}<R_{\emptyset }, \end{array}\right. \end{array}$$

*$T_{\mathrm{decentral}}$ decreases as $\alpha_{\max}$ increases, and $T_{\mathrm{decentral}}$ increases as $R_{u}$ increases if $R_{u}\geq R_{\emptyset }$.*

Due to the complex term $R_{u}$, $T_{\mathrm{decentral}}$ in Theorem 4 is hard to evaluate. Since $T_{\mathrm{decentral}}$ is increasing as $R_{u}$ increases (see Remark 4), substituting the following upper bound of $R_{u}$ into (17) provides an efficient way to evaluate $T_{\mathrm{decentral}}$.

**Corollary** **1.** *For memory size 0≤p≤1, the upper bound of $R_{u}$ is given below:*

- *$\alpha_{\max}=1$ (a shared link):*
(24) $$\begin{array}{cc} R_{u}\leq & \frac{1-p}{p}\left[1-\frac{5}{2}Kp{\left(1-p\right)}^{K-1}-4{\left(1-p\right)}^{K}+\frac{3(1-{\left(1-p\right)}^{K+1})}{(K+1)p}\right]; \end{array}$$
- *$\alpha_{\max}=\left\lfloor \frac{K}{2}\right\rfloor$ (a flexible network):*
(25) $$\begin{array}{cc} \;R_{u}\leq & \frac{K(1-p)}{\left(K-1\right)}\left[1-{\left(1-p\right)}^{K-1}\;-\frac{2/p}{K\;-\;2}\left(1\;-\;{\left(1\;-\;p\right)}^{K}\;-\;Kp{\left(1\;-\;p\right)}^{K\;-\;1}\right)\right].\; \end{array}$$

**Proof.**  See the proof in Appendix C. □

Recall that the transmission delay achieved by the decentralized scheme without D2D communication [2] is equal to $R_{s}$ given in (19). We define the ratio between $T_{\mathrm{decentral}}$ and $R_{s}$ as *decentralized cooperation gain*:

(26) $$\begin{array}{c} G_{\text{decentral,c}}≜\max\left\{\frac{R_{\emptyset }}{R_{s}},\frac{R_{u}}{R_{s}+R_{u}-R_{\emptyset }}\right\}, \end{array}$$

with $G_{\text{decentral,c}}\in \left[0,1\right]$ because of $R_{\emptyset }\leq R_{s}$. Similar to the centralized scenario, this gain arises from the coordination between users in the D2D network. Moreover, we also compare $T_{\mathrm{decentral}}$ with the transmission delay (1−p)/p, achieved by the D2D decentralized coded caching scheme [35], and define the ratio between $R_{s}$ and (1−p)/p as *decentralized parallel gain*:

(27) $$\begin{array}{c} G_{\text{decentral,p}}≜G_{\text{decentral,c}}\cdot \left(1-{\left(1-p\right)}^{K}\right), \end{array}$$

where $G_{\text{decentral,p}}\in \left[0,1\right]$ arises from the parallel transmission between the server and the users.

**Figure 4:**
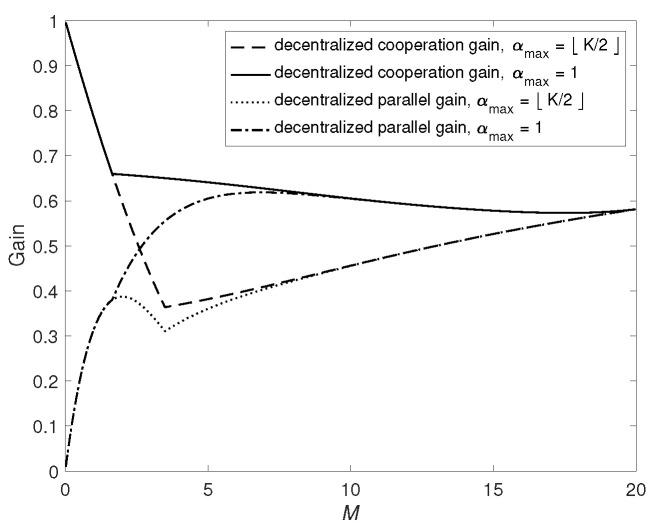
Decentralized cooperation gain and parallel gain when N=20 and K=10.

We plot the decentralized cooperation gain and parallel gain for the two types of D2D networks in Figure 4 when N=20 and K=10. It can be seen that $G_{\text{decentral,c}}$ and $G_{\text{decentral,p}}$ in general are not monotonic functions of *M*. Here $G_{\text{decentral,c}}$ performs in a way similar to $G_{\text{central,c}}$. When *M* is small, the function $G_{\text{decentral,c}}$ is monotonically decreasing from value 1 until reaching the minimum. For larger *M*, the function $G_{\text{decentral,c}}$ turns to monotonically increase with *M*. The reason for this phenomenon is that in the decentralized scenario, when *M* increases, the proportion of subfiles that are not cached by any user and must be sent by the server is decreasing. Thus, there are more subfiles that can be sent parallelly via D2D network as *M* increases. Meanwhile, the decentralized scheme in [2] offers an additional multicasting gain. Therefore, we need to balance these two gains to reduce the transmission delay.

The function $G_{\text{decentral,p}}$ behaves differently as it monotonically increases when *M* is small. After reaching the maximal value, the function $G_{\text{decentral,p}}$ decreases monotonically until meeting the local minimum (The abnormal bend in parallel gain when $\alpha_{\max}=\left\lfloor \frac{K}{2}\right\rfloor$ comes from a balance effect between the $G_{\text{decentral,c}}$ and $1-{\left(1-p\right)}^{K}$ in (27)), then $G_{\text{decentral,p}}$ turns to be a monotonic increasing function for large *M*. Similar to the centralized case, as *M* increases, the impact of parallel transmission among the server and users becomes smaller since more data can be transmitted by the users.

**Theorem** **5.** *Define p≜M/N and $p_{\mathrm{th}}≜1-{\left(\frac{1}{K+1}\right)}^{\frac{1}{K-1}}$, which tends to 0 as K tends to infinity. For memory size 0≤M≤N,*

- *if $\alpha_{\max}=1$ (shared link), then*
$$\frac{T_{\mathrm{decentral}}}{T^{*}}\leq 24;$$
- *if $\alpha_{\max}=\left\lfloor \frac{K}{2}\right\rfloor$, then*
$$\begin{array}{c} \frac{T_{\mathrm{decentral}}}{T^{*}}\leq \left\{\begin{array}{ccc} & \max\left\{6,2K{\left(\frac{2K}{2K+1}\right)}^{K-1}\right\}, & p<p_{\mathrm{th}},\;\\ & 6, & p\geq p_{\mathrm{th}}.\; \end{array}\right. \end{array}$$

**Proof.**  See the proof in Appendix D. □

**Figure 5:**
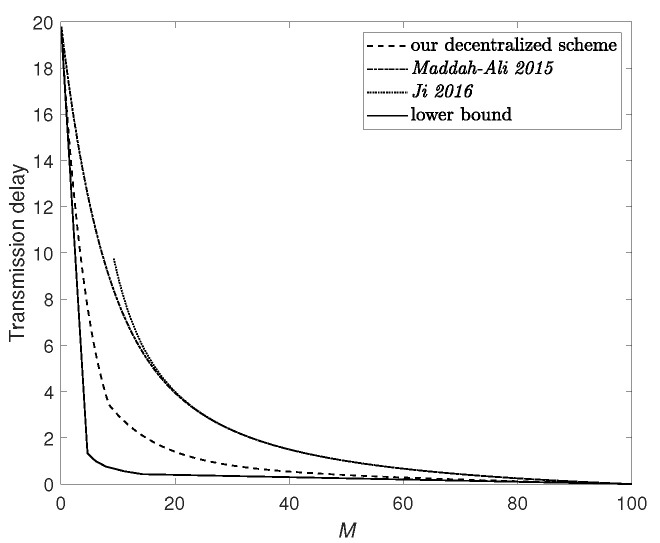
Transmission delay when N=100, K=20 and $\alpha_{\max}=3$. The upper bounds are achieved under the decentralized random caching scenario.

Figure 5 plots the lower bound in (9) and upper bounds achieved by various decentralized coded caching schemes, including our scheme, the scheme *Maddah-Ali 2015* in [2] which considers the case without D2D communication, and the scheme *Ji 2016* in [35] which considers the case without server.

## 4. Coding Scheme under Centralized Data Placement

In this section, we describe a novel centralized coded caching scheme for arbitrary *K*, *N* and *M* such that t=KM/N is a positive integer. The scheme can be extended to the general case 1≤t≤K by following the same approach as in [1].

We first use an illustrative example to show how we form D2D communication groups, split files and deliver data, and then present our generalized centralized coding caching scheme.

### 4.1. An Illustrative Example

Consider a network consisting of K=6 users with cache size M=4, and a library of N=6 files. Thus, t=KM/N=4. Divide all six users into two groups of equal size, and choose an integer $L_{1}=2$ that guarantees KK−1tL1min{α(⌊K/α⌋−1),t} to be an integer. (According to (11) and (29), one optimal choice could be (α=1, $L_{1}=4$, λ=5/9), here we choose (α=2, $L_{1}=2$, λ=1/3) for simplicity, and also in order to demonstrate that even with a suboptimal choice, our scheme still outperforms that in [1,35]). Split each file $W_{n}$, for n=1,…,N, into 364=45 subfiles:

$$W_{n}=(W_{n,T}^{l}:l\in \left[3\right],T\subset \left[6\right],|T|=4).$$

We list all the requested subfiles uncached by all users as follows: for l=1,2,3,

$$\begin{array}{cc} \; & W_{d_{1},\left\{2,3,4,5\right\}}^{l},W_{d_{1},\left\{2,3,4,6\right\}}^{l},W_{d_{1},\left\{2,3,5,6\right\}}^{l},W_{d_{1},\left\{2,4,5,6\right\}}^{l},W_{d_{1},\left\{3,4,5,6\right\}}^{l};\\ & W_{d_{2},\left\{1,3,4,5\right\}}^{l},W_{d_{2},\left\{1,3,4,6\right\}}^{l},W_{d_{2},\left\{1,3,5,6\right\}}^{l},W_{d_{2},\left\{1,4,5,6\right\}}^{l},W_{d_{2},\left\{3,4,5,6\right\}}^{l};\\ & W_{d_{3},\left\{1,2,4,5\right\}}^{l},W_{d_{3},\left\{1,2,4,6\right\}}^{l},W_{d_{3},\left\{1,2,5,6\right\}}^{l},W_{d_{3},\left\{1,4,5,6\right\}}^{l},W_{d_{3},\left\{2,4,5,6\right\}}^{l};\\ & W_{d_{4},\left\{1,2,3,5\right\}}^{l},W_{d_{4},\left\{1,2,3,6\right\}}^{l},W_{d_{4},\left\{1,2,5,6\right\}}^{l},W_{d_{4},\left\{1,3,5,6\right\}}^{l},W_{d_{4},\left\{2,3,5,6\right\}}^{l};\\ & W_{d_{5},\left\{1,2,3,4\right\}}^{l},W_{d_{5},\left\{1,2,3,6\right\}}^{l},W_{d_{5},\left\{1,2,4,6\right\}}^{l},W_{d_{5},\left\{1,3,4,6\right\}}^{l},W_{d_{5},\left\{2,3,4,6\right\}}^{l};\\ & W_{d_{6},\left\{1,2,3,4\right\}}^{l},W_{d_{6},\left\{1,2,3,5\right\}}^{l},W_{d_{6},\left\{1,2,4,5\right\}}^{l},W_{d_{6},\left\{1,3,4,5\right\}}^{l},W_{d_{6},\left\{2,3,4,5\right\}}^{l}. \end{array}$$

The users can finish the transmission in different partitions. Table 1 shows the transmission in four different partitions over the D2D network.

In Table 1, all users first send XOR symbols with superscript l=1. Please note that the subfiles $W_{d_{2},\left\{1,3,4,5\right\}}^{1}$ and $W_{d_{5},\left\{1,2,4,6\right\}}^{1}$ are not delivered at the beginning since KK−1tα(⌊K/α⌋−1) is not an integer. Similarly, for subfiles with l=2, $W_{d_{3},\left\{1,2,5,6\right\}}^{2}$ and $W_{d_{4},\left\{2,3,5,6\right\}}^{2}$ remain to be sent to user 3 and 4. In the last transmission, user 1 delivers the XOR message $W_{d_{3},\left\{1,2,5,6\right\}}^{2}⊕W_{d_{2},\left\{1,3,4,5\right\}}^{1}$ to user 2 and 3, and user 6 multicasts $W_{d_{5},\left\{1,2,4,6\right\}}^{1}⊕W_{d_{4},\left\{2,3,5,6\right\}}^{2}$ to user 5 and 6. The transmission rate in the D2D network is $R_{2}=\frac{1}{3}.$

For the remaining subfiles with superscript l=3, the server delivers them in the same way as in [1]. Specifically, it sends symbols ${⊕}_{k\in S}W_{d_{k},S\setminus \left\{k\right\}}^{3}$, for all S⊆[K]:|S|=5. Thus, the rate sent by the server is $R_{1}=\frac{2}{15}$, and the transmission delay $T_{\mathrm{central}}=\max\left\{R_{1},R_{2}\right\}=\frac{1}{3}$, which is less than the delay achieved by the coded caching schemes for the broadcast network [1] and the D2D communication [35], respectively.

### 4.2. The Generalized Centralized Coding Caching Scheme

In the placement phase, each file is first split into Kt subfiles of equal size. More specifically, split $W_{n}$ into subfiles as follows: $W_{n}=\left(W_{n,T}:T\subset \left[K\right],\left|T\right|=t\right)$. User *k* caches all the subfiles if k∈T for all n=1,...,N, occupying the cache memory of MF bits. Then split each subfile $W_{n,T}$ into two mini-files as $W_{n,T}=\left(W_{n,T}^{s},W_{n,T}^{u}\right)$, where

(28) |Wn,Ts|=λ·|Wn,T|=λ·FKt,|Wn,Tu|=(1−λ)·|Wn,T|=(1−λ)·FKt,

with

(29) $$\begin{array}{c} \lambda =\frac{1+t}{\alpha \min\{\left\lfloor \frac{K}{\alpha }\right\rfloor -1,t\}+1+t}. \end{array}$$

Here, the mini-file $W_{n,T}^{s}$ and $W_{n,T}^{u}$ will be sent by the server and users, respectively. For each mini-file $W_{n,T}^{u}$, split it into $L_{1}$ pico-files of equal size (1−λ)·FL1Kt, i.e., $W_{n,T}^{u}=\left(W_{n,T}^{u,1},\ldots ,W_{n,T}^{u,L_{1}}\right),$ where $L_{1}$ satisfies

(30) K·K−1t·L1αmin{⌊Kα⌋−1,t}∈Z+.

As we will see later, condition (29) ensures that communication loads can be optimally allocated between the server and the users, and (30) ensures that the number of subfiles is large enough to maximize multicast gain for the transmission in the D2D network.

In the delivery phase, each user *k* requests file $W_{d_{k}}$. The request vector $d=(d_{1},d_{2},\ldots ,d_{K})$ is informed by the server and all users. Please note that different parts of file $W_{d_{k}}$ have been stored in the user cache memories, and thus the uncached parts of $W_{d_{k}}$ can be sent both by the server and users. Subfiles

$$\left(W_{d_{k},T}^{u,1},\ldots ,W_{d_{k},T}^{u,L_{1}}:T\subset \left[K\right],\left|T\right|=t,k\notin T\right)$$

are requested by user *k* and will be sent by the users via the D2D network. Subfiles

$$\left(W_{d_{k},T}^{s}:T\subset \left[K\right],\left|T\right|=t,k\notin T\right)$$

are requested by user *k* and will be sent by the server via the broadcast network.

First consider the subfiles sent by the users. Partition the *K* users into α groups of equal size:

$$G_{1},\ldots ,G_{\alpha },$$

where for i,j=1,…,α, $G_{i}\subseteq \left[K\right]:\left|G_{i}\right|=\left\lfloor K/\alpha \right\rfloor$, and $G_{i}\cap G_{j}=\emptyset$, if i≠j. In each group $G_{i}$, one of ⌊K/α⌋ users plays the role of server and sends symbols based on its cached contents to the remaining (⌊K/α⌋−1) users within the group.

Focus on a group $G_{i}$ and a set S⊂[K]:|S|=t+1. If $G_{i}\subseteq S$, then all nodes in $G_{i}$ share subfiles

$$(W_{n,T}^{u,l}:l\in \left[L_{1}\right],n\in \left[N\right],G_{i}\subseteq T,|T|=t).$$

In this case, user $k\in G_{i}$ sends XOR symbols that contains the requested subfiles useful to all remaining ⌊K/α⌋−1 users in $G_{i}$, i.e., ${⊕}_{j\in G_{i}\setminus \left\{k\right\}}W_{d_{j},S\setminus \left\{j\right\}}^{u,l\left(k,G_{i},S\right)},$ where $l\left(k,G_{i},S\right)\in \left[L_{1}\right]$ is a function of $\left(k,G_{i},S\right)$ which avoids redundant transmission of any fragments.

If $S\subseteq G_{i}$, then the nodes in S share subfiles

$$(W_{n,T}^{u,l}:l\in \left[L_{1}\right],n\in \left[N\right],T\subset S,|T|=t).$$

In this case, user k∈S sends an XOR symbol that contains the requested subfiles for all remaining t−1 users in S, i.e., ${⊕}_{j\in S\setminus \{k\}}W_{d_{j},S\setminus \left\{j\right\}}^{u,l\left(k,G_{i},S\right)}$. Other groups perform the similar steps and concurrently deliver the remaining requested subfiles to other users.

By changing group partition and performing the delivery strategy described above, we can send all the requested subfiles

(31) $$\begin{array}{c} (W_{d_{k},T}^{u,1},\ldots ,W_{d_{k},T}^{u,L_{1}}{:T\subset \left[K\right],|T|=t,k\notin T)}_{k=1}^{K} \end{array}$$

to the users.

Since α groups send signals in a parallel manner (α users can concurrently deliver contents), and each user in a group delivers a symbol containing min{⌊K/α⌋−1,t} non-repeating pico-files requested by other users, in order to send all requested subfiles in (31), we need to send in total

(32) K·K−1t·L1αmin{⌊Kα⌋−1,t}

XOR symbols, each of size 1−λKtF bits. Notice that $L_{1}$ is chosen according to (30), ensuring that (32) equals to an integer. Thus, we obtain $R_{2}$ as

(33) R2=KL1·K−1tαmin{⌊Kα⌋−1,t}·1−λL1Kt=K1−MN11+t+αmin{⌊Kα⌋−1,t},

where the last equality holds by (29).

Now consider the delivery of the subfiles sent by the server. Apply the delivery strategy as in [1], i.e., the server broadcasts

$${⊕}_{k\in S}W_{d_{k},S\setminus \left\{k\right\}}^{s}$$

to all users, for all S⊆[K]:|S|=t+1. We obtain the transmission rate of the server

(34) $$\begin{array}{cc} R_{1}= & \lambda \cdot K\left(1-\frac{M}{N}\right)\cdot \frac{1}{1+t}\\ = & K\left(1\;-\;\frac{M}{N}\right)\frac{1}{1\;+\;t\;+\;\alpha \min\{\left\lfloor \frac{K}{\alpha }\right\rfloor \;-\;1,t\}}. \end{array}$$

From (33) and (34), we can see that the choice λ in (29) guarantees equal communication loads at the server and users. Since the server and users transmit the signals simultaneously, the transmission delay of the whole network is the maximum between $R_{1}$ and $R_{2}$, i.e., $T_{\mathrm{central}}=\max\left\{R_{1},R_{2}\right\}=\frac{K(1\;-\;M/N)}{1+t+\alpha \min\{\lfloor K/\alpha \rfloor -1,t\}}$, for some $\alpha \in [\alpha_{\max}]$.

## 5. Coding Scheme under Decentralized Data Placement

In this section, we present a novel decentralized coded caching scheme for joint broadcast network and D2D network. The decentralized scenario is much more complicated than the centralized scenario, since each subfile can be stored by s=1,2,…,K users, leading to a dynamic file-splitting and communication strategy in the D2D network. We first use an illustrative example to demonstrate how we form D2D communication groups, split data and deliver data, and then present our generalized coding caching scheme.

### 5.1. An Illustrative Example

Consider a joint broadcast and D2D network consisting of K=7 users. When using the decentralized data placement strategy, the subfiles cached by user *k* can be written as

(35) $$\begin{array}{c} \left(W_{n,T}:n\in \left[N\right],k\in T,T\subseteq \left[7\right]\right). \end{array}$$

We focus on the delivery of subfiles $W_{n,T}:n\in \left[N\right],k\in T,\left|T\right|=s=4$, i.e., each subfile is stored by s=4 users. A similar process can be applied to deliver other subfiles with respect to s∈[K]∖{4}.

To allocate communication loads between the server and users, we divide each subfile into two mini-files $W_{n,T}=\left(W_{n,T}^{s},W_{n,T}^{u}\right)$, where mini-files $\left\{W_{n,T}^{s}\right\}$ and $\left\{W_{n,T}^{u}\right\}$ will be sent by the server and users, respectively. To reduce the transmission delay, the size of $W_{n,T}^{s}$ and $W_{n,T}^{u}$ need to be chosen properly such that $R_{1}=R_{2}$, i.e., the transmission rate of the server and users are equal; see (37) and (39) ahead.

Divide all the users into two non-intersecting groups $\left(G_{1}^{r},G_{2}^{r}\right)$, for r∈[35] which satisfies

$$G_{1}^{r}\subset \left[K\right],G_{2}^{r}\subset \left[K\right],|G_{1}^{r}\left|=4,\right|G_{2}^{r}|=3,G_{1}^{r}\cap G_{2}^{r}=\emptyset .$$

There are 74=35 kinds of partitions in total, thus r∈[35]. Please note that for any user $k\in G_{i}^{r}$, $|G_{i}^{r}|-1$ of its requested mini-files are already cached by the rest users in $G_{i}^{r}$, for i=1,2.

To avoid repetitive transmission of any mini-file, each mini-file in

$$\left(W_{d_{k},T\setminus \left\{k\right\}}^{u}:T\subseteq \left[7\right],k\in \left[7\right]\right)$$

is divided into non-overlapping pico-files $W_{d_{k},T\setminus \left\{k\right\}}^{u_{1}}$ and $W_{d_{k},T\setminus \left\{k\right\}}^{u_{2}}$, i.e.,

$$\begin{array}{c} W_{d_{k},T\setminus \left\{k\right\}}^{u}=\left(W_{d_{k},T\setminus \left\{k\right\}}^{u_{1}},W_{d_{k},T\setminus \left\{k\right\}}^{u_{2}}\right). \end{array}$$

The sizes of $W_{n,T}^{u_{1}}$ and $W_{n,T}^{u_{2}}$ need to be chosen properly to have equal transmission rate of group $G_{1}^{r}$ and $G_{2}^{r}$; see (51) and (52) ahead.

To allocate communication loads between the two different types of groups, split each $W_{d_{k},T\setminus \left\{k\right\}}^{u_{1}}$ and $W_{d_{k},T\setminus \left\{k\right\}}^{u_{2}}$ into 3 and two equal fragments, respectively, e.g.,

$$\begin{array}{ccc} & & W_{d_{2},\left\{1,3,4\right\}}^{u_{1}}=\left(W_{d_{2},\left\{1,3,4\right\}}^{u_{1},1},W_{d_{2},\left\{1,3,4\right\}}^{u_{1},2},W_{d_{2},\left\{1,3,4\right\}}^{u_{1},3}\right),\\ & & W_{d_{2},\left\{1,3,4\right\}}^{u_{2}}=\left(W_{d_{2},\left\{1,3,4\right\}}^{u_{2},1},W_{d_{2},\left\{1,3,4\right\}}^{u_{2},2}\right). \end{array}$$

During the delivery phase, in each round, one user in each group produces and multicasts an XOR symbol to all other users in the same group, as shown in Table 2.

Please note that in this example, each group only appears one time among all partitions. However, for some other values of *s*, each group could appear multiple times in different partitions. For example, when s=2, group {1,2} appears in both partitions {{1,2},{3,4},{5,6,7}} and {{1,2},{3,5},{4,6,7}}. To reduce the transmission delay, one should balance communication loads between all groups, and between the server and users as well.

### 5.2. The Generalized Decentralized Coded Caching Scheme

In the placement phase, each user *k* applies the caching function to map a subset of $\frac{MF}{N}$ bits of file $W_{n},n=1,...,N,$ into its cache memory at random: $W_{n}=\left(W_{n,T}:T\subseteq \left[K\right]\right).$ The subfiles cached by user *k* can be written as $\left(W_{n,T}:n\in \left[N\right],k\in T,T\subseteq \left[K\right]\right).$ When the size of file *F* is sufficiently large, by the law of large numbers, the subfile size with high probability can be written by

(36) $$\begin{array}{c} |W_{n,T}|\approx p^{\left|T\right|}{\left(1-p\right)}^{K-|T|}. \end{array}$$

The delivery procedure can be characterized into three different levels: allocating communication loads between the server and user, inner-group coding (i.e., transmission in each group) and parallel delivery among groups.

#### 5.2.1. Allocating Communication Loads between the Server and User

To allocate communication loads between the server and users, split each subfile $W_{n,T}$, for T⊆[K]:T≠∅, into two non-overlapping mini-files

$$W_{n,T}=\left(W_{n,T}^{s},W_{n,T}^{u}\right),$$

where

(37) $$\begin{array}{c} |W_{n,T}^{s}\left|=\lambda \cdot \right|W_{n,T}|,\\ |W_{n,T}^{u}\left|=\left(1-\lambda \right)\cdot \right|W_{n,T}|, \end{array}$$

and λ is a design parameter whose value is determined in Remark 5.

Mini-files $\left(W_{d_{k},T\setminus \left\{k\right\}}^{s}:k\in \left[K\right]\right)$ will be sent by the server using the decentralized coded caching scheme for the broadcast network [2], leading to the transmission delay

(38) $$\begin{array}{c} \lambda R_{s}=\lambda \frac{1-M/N}{M/N}\left(1-{\left(1-\frac{M}{N}\right)}^{K}\right), \end{array}$$

where $R_{s}$ is defined in (19).

Mini-files $\left(W_{d_{k},T\setminus \left\{k\right\}}^{u}:k\in \left[K\right]\right)$ will be sent by users using *parallel user delivery* described in Section 5.2.3. The corresponding transmission rate is

(39) $$\begin{array}{c} R_{2}=\left(1-\lambda \right)R_{u}, \end{array}$$

where $R_{u}$ represents the transmission bits sent by each user normalized by *F*.

Since subfile $W_{d_{k},\emptyset }$ is not cached by any user and must be sent exclusively from the server, the corresponding transmission delay for sending $\left(W_{d_{k},\emptyset }:k\in \left[K\right]\right)$ is

(40) $$\begin{array}{c} R_{\emptyset }=K{\left(1-\frac{M}{N}\right)}^{K}, \end{array}$$

where $R_{\emptyset }$ coincides with the definition in (18).

By (38)–(40), we have

(41) $$\begin{array}{c} R_{1}=R_{\emptyset }+\lambda R_{s},\;\;R_{2}=\left(1-\lambda \right)R_{u}. \end{array}$$

According to (8), we have $T_{\mathrm{decentral}}=\max\left\{R_{1},R_{2}\right\}$.

**Remark** **5** (Choice of λ).*The parameter λ is chosen such that $T_{\mathrm{decentral}}$ is minimized. If $R_{u}<R_{\emptyset }$, then the inequality $R_{2}\leq R_{1}$ always holds and $T_{\mathrm{decentral}}$ reaches the minimum $T_{\mathrm{decentral}}=R_{\emptyset }$ with λ=0. If $R_{u}\geq R_{\emptyset }$, solving $R_{1}=R_{2}$ yields $\lambda =\frac{R_{u}-R_{\emptyset }}{R_{s}+R_{u}}$ and $T_{\mathrm{decentral}}=\frac{R_{s}R_{u}}{R_{s}+R_{u}-R_{\emptyset }}.$*

#### 5.2.2. Inner-Group Coding

Given parameters (s,G,i,γ) where s∈[K−1], G⊆[K], $i\in \{u,u_{1},u_{2}\}$ with indicators $u,u_{1},u_{2}$ described in (37) and (51), and $\gamma \in Z^{+}$, we present how to successfully deliver

$$\left(W_{d_{k},S\setminus \left\{k\right\}}^{i}:\forall S\subseteq \left[K\right],|S|=s,G\subseteq S\right)$$

to every user k∈G via D2D communication.

Split each $W_{d_{k},S\setminus \left\{k\right\}}^{i}$ into (|G|−1)γ non-overlapping fragments of equal size, i.e.,

(42) $$\begin{array}{c} W_{d_{k},S\setminus \left\{k\right\}}^{i}=\left(W_{d_{k},S\setminus \left\{k\right\}}^{i,l}:l\in \left[(|G\right|-1)\gamma ]\right), \end{array}$$

and each user k∈G takes turn to broadcast XOR symbol

(43) $$\begin{array}{c} X_{k,G,s}^{i}≜{⊕}_{j\in G\setminus \{k\}}W_{d_{j},S\setminus \left\{j\right\}}^{i,l\left(j,G,S\right)}, \end{array}$$

where l(k,G,S)∈[(|G|−1)γ] is a function of (k,G,S) which avoids redundant transmission of any fragments. The XOR symbol $X_{k,G,s}^{i}$ will be received and decoded by the remaining users in G.

For each group G, inner-group coding encodes in total K−|G|s−|G| of $W_{d_{k},S\setminus \left\{k\right\}}^{i}$, and each XOR symbol $X_{k,G,s}^{i}$ in (43) contains fragments required by |G|−1 users in G.

#### 5.2.3. Parallel Delivery among Groups

The parallel user delivery consists of (K−1) rounds characterized by s=2,⋯,K. In each round *s*, mini-files

$$\left(W_{d_{k},T\setminus \left\{k\right\}}^{u}:\forall T\subseteq \left[K\right],|T|=s,k\in \left[K\right]\right)$$

are recovered through D2D communication.

The key idea is to partition *K* users into $\left\lceil \frac{K}{s}\right\rceil$ groups for each communication round s∈{2,...,K}, and let each group perform the D2D coded caching scheme [35] to exchange information. If (Kmods)≠0, there will be $\left\lfloor \frac{K}{s}\right\rfloor$ numbers of groups of the same size *s*, and an *abnormal* group of size (Kmods), leading to an asymmetric caching setup. We optimally allocate the communication loads between the two types of groups, and between the broadcast network and D2D network as well.

Based on *K*, *s* and $\alpha_{\max}$, the delivery strategy in the D2D network is divided into 3 cases:

- Case 1: $\left\lceil \frac{K}{s}\right\rceil >\alpha_{\max}$. In this case, $\alpha_{\max}$ users are allowed to send data simultaneously. Select $s\cdot \alpha_{\max}$ users from all users and divide them into $\alpha_{\max}$ groups of equal size *s*. The total number of such kinds of partition is
  (44) β1≜KsK−ss⋯K−s(αmax−1)sαmax!.
  In each partition, $\alpha_{\max}$ users, selected from $\alpha_{\max}$ groups, respectively, send data in parallel via the D2D network.
- Case 2: $\left\lceil \frac{K}{s}\right\rceil \leq \alpha_{\max}$ and (Kmods)<2. In this case, choose $(\left\lfloor \frac{K}{s}\right\rfloor -1)s$ users from all users and partition them into $\left(\left\lfloor \frac{K}{s}\right\rfloor -1\right)$ groups of equal size *s*. The total number of such kind partition is
  (45) β2≜KsK−ss⋯K−s(⌊Ks⌋−1)s⌊Ks⌋!.
  In each partition, $\left(\left\lfloor \frac{K}{s}\right\rfloor -1\right)$ users selected from $\left(\left\lfloor \frac{K}{s}\right\rfloor -1\right)$ groups of equal size *s*, respectively, together with an extra user selected from the *abnormal* group of size $K-s(\left\lfloor \frac{K}{s}\right\rfloor -1)$ send data in parallel via the D2D network.
- Case 3: $\left\lceil \frac{K}{s}\right\rceil \leq \alpha_{\max}$ and (Kmods)≥2. In this case, every *s* users form a group, resulting in $\left\lfloor \frac{K}{s}\right\rfloor$ groups consisting of $s\lfloor \frac{K}{s}\rfloor$ users. The remaining (Kmods) users form an *abnormal* group. The total number of such kind of partition is
  (46) $$\begin{array}{c} \beta_{3}=\beta_{2}. \end{array}$$
  In each partition, $\left\lfloor \frac{K}{s}\right\rfloor$ users selected from $\left\lfloor \frac{K}{s}\right\rfloor$ groups of equal size *s*, respectively, together with an extra user selected from the abnormal group of size (Kmods) send data in parallel via the D2D network.

Thus, the exact number of users who parallelly send signals can be written as follows:

(47) $$\alpha_{D}=\;\left\{\begin{array}{ccc} \; & \alpha_{\max},\; & \mathrm{case}\;1,\\ \; & \lfloor \frac{K}{s}\rfloor ,\; & \mathrm{case}\;2,\\ \; & \lceil \frac{K}{s}\rceil ,\; & \mathrm{case}\;3. \end{array}\right.$$

Please note that each group G re-appears

(48) NG≜K−ss⋯K−s·(αD−1)s(αD−1)!

times among $\left[\beta_{c}\right]$ partitions.

Now we present the decentralized scheme for these three cases as follows.

*Case 1* ($\left\lceil \frac{K}{s}\right\rceil >\alpha_{\max}$): Consider a partition $r\in [\beta_{1}]$, denoted by

$$G_{1}^{r},\ldots ,G_{\alpha_{D}}^{r},$$

where $|G_{i}^{r}|=s$ and $G_{i}^{r}\cap G_{j}^{r}=\emptyset$, $\forall i,j\in [\alpha_{D}]$ and i≠j.

Since each group $G_{i}^{r}$ re-appears $N_{G_{i}^{r}}$ times among $\left[\beta_{1}\right]$ partitions, and $\left(|G_{i}^{r}|-1\right)$ users take turns to broadcast XOR symbols (43) in each group $G_{i}^{r}$, in order to guarantee that each group can send a unique fragment without repetition, we split each mini-file $W_{d_{k},S\setminus \left\{k\right\}}^{u}$ into $\left(\right|G_{i}^{r}\left|-1\right)N_{G_{i}^{r}}$ fragments of equal size.

Each group $G_{i}^{r}$, for $r\in [\beta_{1}]$ and $i\in [\alpha_{D}]$, performs inner-group coding (see Section 5.2.2) with parameters

$$(s,G_{i}^{r},u,N_{G_{i}^{r}}),$$

for all *s* satisfying $\left\lceil \frac{K}{s}\right\rceil >\alpha_{\max}$. For each round *r*, all groups $G_{1}^{r},\ldots ,G_{\alpha_{D}}^{r}$ parallelly send XOR symbols containing $|G_{i}^{r}|-1$ fragments required by other users of its group. By the fact that the partitioned groups traverse every set T, i.e.,

$$T\subseteq {\left\{G_{1}^{r}\cup \ldots \cup G_{\alpha_{D}}^{r}\right\}}_{r=1}^{\beta_{1}},\forall T\subseteq \left[K\right]:\left|T\right|=s,$$

and since inner-group coding enables each group $G_{i}^{r}$ to recover

$$(W_{d_{k},S\setminus \left\{k\right\}}^{u}:\forall S\subseteq \left[K\right],|S|=s,G_{i}^{r}\subseteq S,k\in \left[K\right]),$$

we can recover all required mini-files

$$(W_{d_{k},T\setminus \left\{k\right\}}^{u}:\forall T\subseteq \left[K\right],|T|=s,k\in \left[K\right]).$$

The transmission delay of Case 1 in round *s* is thus

(49) Rcase1u(s)≜∑r∈[β1]∑k∈Gir|Xk,Gir,su|=(a)KK−1s−1αD(s−1)|Wdk,T∖{k}u|=KK−1s−1αmax(s−1)(1−λ)ps−1(1−p)K−s+1,

where (a) follows by (44) and (48).

*Case 2* ($\left\lceil \frac{K}{s}\right\rceil \leq \alpha_{\max}$ and (Kmods)<2): We apply the same delivery procedure as Case 1, except that $\beta_{1}$ is replaced by $\beta_{2}$ and $\alpha_{D}=\left\lfloor \frac{K}{s}\right\rfloor$. Thus, the transmission delay in round *s* is

(50) Rcase2u(s)=KK−1s−1αD(s−1)|Wdk,T∖{k}u|=KK−1s−1⌊Ks⌋(s−1)(1−λ)ps−1(1−p)K−s+1.

*Case 3* ( $\left\lceil \frac{K}{s}\right\rceil \leq \alpha_{\max}$ and (Kmods)≥2): Consider a partition $r\in [\beta_{3}]$, denoted as

$$G_{1}^{r},\ldots ,G_{\alpha_{D}}^{r},$$

where $G_{i}^{r}\subseteq \left[K\right]$, $G_{i}^{r}\cap G_{j}^{r}=\emptyset$, $\forall i,j\in [\alpha_{D}-1]$ and i≠j and $G_{\alpha_{D}}^{r}=\left[K\right]\setminus \left(\cup_{i=1}^{\alpha_{D}-1}G_{i}^{r}\right)$ with $|G_{i}^{r}\left|=s,\;\right|G_{\alpha_{D}}^{r}|=\left(K\;\mathrm{mod}\;s\right).$

Since group $G_{i}^{r}:i\in \left[\alpha_{D}-1\right]$ and $G_{\alpha_{D}}^{r}$ have different group sizes, we further split each mini-file $W_{d_{k},T\setminus \left\{k\right\}}^{u}$ into two non-overlapping fragments such that

(51) $$\begin{array}{c} |W_{d_{k},T\setminus \left\{k\right\}}^{u_{1}}|=\lambda_{2}\left|W_{d_{k},T\setminus \left\{k\right\}}^{u}\right|,\\ |W_{d_{k},T\setminus \left\{k\right\}}^{u_{2}}|=\left(1-\lambda_{2}\right)\left|W_{d_{k},T\setminus \left\{k\right\}}^{u}\right|, \end{array}$$

where $\lambda_{2}\in \left[0,1\right]$ is a designed parameter satisfying (52).

Split each mini-file $W_{d_{k},S\setminus \left\{k\right\}}^{u_{1}}$ and $W_{d_{k},S\setminus \left\{k\right\}}^{u_{2}}$ into fragments of equal size:

Wdk,S∖{k}u1=Wdk,S∖{k}u1,l:l∈[(s−1)NGir],Wdk,S∖{k}u2=Wdk,S∖{k}u2,l:l∈|GαDr|−1s−1|GαDr|−1NGir.

Following the similar encoding operation in (43), group $G_{i}^{r}:i\in \left[\alpha_{D}-1\right]$ and group $G_{\alpha_{D}}^{r}$ send the following XOR symbols, respectively:

$$\begin{array}{ccc} & & {\left(X_{k,G_{i}^{r},s}^{u_{1}}:k\in G_{i}^{r}\right)}_{i=1}^{\left(\alpha_{D}-1\right)},\;\left(X_{k,G_{\alpha_{D}}^{r},s}^{u_{2}}:k\in G_{\alpha_{D}}^{r}\right). \end{array}$$

For each s∈{2,…,K}, the transmission delay for sending the XOR symbols above by group $G_{i}^{r}:i\in \left[\alpha_{D}-1\right]$ and group $G_{\left\lceil \frac{K}{s}\right\rceil }^{r}$ can be written as

Rcase3u1(s)=λ2KK−1s−1(αD−1)(s−1)·|Wdk,T∖{k}u|,Rcase3u2(s)=(1−λ2)KK−1s−1(Kmods)−1·|Wdk,T∖{k}u|,

respectively. Since $G_{i}:i\in \left[\left\lfloor \frac{K}{s}\right\rfloor \right]$ and group $G_{\left\lceil \frac{K}{s}\right\rceil }$ can send signals in parallel, by letting

(52) $$\begin{array}{c} R_{\mathrm{case}3}^{u_{1}}\left(s\right)=R_{\mathrm{case}3}^{u_{2}}\left(s\right), \end{array}$$

we eliminate the parameter $\lambda_{2}$ and obtain the balanced transmission delay at users for Case 3:

(53) Rcase3u(s)≜KK−1s−1K−1−⌊Ks⌋(1−λ)ps−1(1−p)K−s+1.

**Remark** **6.** *The condition $\left\lceil \frac{K}{s}\right\rceil >\alpha_{\max}$ in Case 1 implies that $s\leq \lceil \frac{K}{\alpha_{\max}}\rceil -1$. In this regime, the transmission delay is given in (49). If $s\geq \lceil \frac{K}{\alpha_{\max}}\rceil -1$ and (Kmods)<2, scheme for Case 2 starts to work and the transmission delay is given in (50); If $s\geq \lceil \frac{K}{\alpha_{\max}}\rceil -1$ and (Kmods)≥2, scheme for Case 3 starts to work and the transmission delay is given in (53).*

In each round s∈{2,…,K}, all requested mini-files can be recovered by the delivery strategies above. By Remark 6, the transmission delay in the D2D network is

(54) R2=(1−λ)1αmax∑s=2⌈Kαmax⌉−1sKss−1ps−1(1−p)K−s+1+(1−λ)∑s=⌈Kαmax⌉KKK−1s−1f(K,s)ps−1(1−p)K−s+1=(1−λ)Ru,

where $R_{u}$ is defined in (20) and

(55) $$\begin{array}{c} f\left(K,s\right)≜\left\{\begin{array}{ccc} & \left\lfloor \frac{K}{s}\right\rfloor \left(s-1\right), & (K\;mod\;s)<2,\\ & K-1-\lfloor K/s\rfloor , & (K\;mod\;s)\geq 2. \end{array}\right. \end{array}$$

## 6. Conclusions

In this paper, we considered a cache-aided communication via joint broadcast network with a D2D network. Two novel coded caching schemes were proposed for centralized and decentralized data placement settings, respectively. Both schemes achieve a parallel gain and a cooperation gain by efficiently exploiting communication opportunities in the broadcast and D2D networks, and optimally allocating communication loads between the server and users. Furthermore, we showed that in the centralized case, letting too many users parallelly send information could be harmful. The information theoretic converse bounds were established, with which we proved that the centralized scheme achieves the optimal transmission delay within a constant multiplicative gap in all regimes, and the decentralized scheme is also order-optimal when the cache size of each user is larger than a small threshold which tends to zero as the number of users tends to infinity. Our work indicates that combining the cache-aided broadcast network with the cache-aided D2D network can greatly reduce the transmission latency.

## Figures and Tables

**Table 1:** Subfiles sent by users in different partition, l=1,2.

{1,2,3}	{4,5,6}
user 2: $W_{d_{1},\left\{2,3,4,5\right\}}^{1}\;⊕\;W_{d_{3},\left\{1,2,4,5\right\}}^{1}$	user 5: $W_{d_{4},\left\{2,3,5,6\right\}}^{1}\;⊕\;W_{d_{6},\left\{2,3,4,5\right\}}^{1}$
user 2: $W_{d_{1},\left\{2,3,4,6\right\}}^{1}\;⊕\;W_{d_{3},\left\{1,2,4,6\right\}}^{1}$	user 5: $W_{d_{4},\left\{1,2,5,6\right\}}^{1}\;⊕\;W_{d_{6},\left\{1,2,4,5\right\}}^{1}$
user 1: $W_{d_{2},\left\{1,3,4,6\right\}}^{1}\;⊕\;W_{d_{3},\left\{1,2,5,6\right\}}^{1}$	user 4: $W_{d_{5},\left\{2,3,4,6\right\}}^{1}\;⊕\;W_{d_{6},\left\{1,3,4,5\right\}}^{1}$
user 3: $W_{d_{1},\left\{2,3,5,6\right\}}^{1}\;⊕\;W_{d_{2},\left\{1,3,5,6\right\}}^{1}$	user 6: $W_{d_{4},\left\{1,3,5,6\right\}}^{1}\;⊕\;W_{d_{5},\left\{1,3,4,6\right\}}^{1}$
{1,2,4}	{3,5,6}
user 2: $W_{d_{1},\left\{2,4,5,6\right\}}^{l}\;⊕\;W_{d_{4},\left\{1,2,3,5\right\}}^{l}$	user 5: $W_{d_{3},\left\{1,4,5,6\right\}}^{l}\;⊕\;W_{d_{6},\left\{1,2,3,5\right\}}^{l}$
{1,4,6}	{2,3,5}
user 6: $W_{d_{1},\left\{3,4,5,6\right\}}^{l}\;⊕\;W_{d_{4},\left\{1,2,3,6\right\}}^{l}$	user 3: $W_{d_{2},\left\{3,4,5,6\right\}}^{l}\;⊕\;W_{d_{5},\left\{1,2,3,4\right\}}^{l}$
{1,2,5}	{3,4,6}
user 1: $W_{d_{2},\left\{1,4,5,6\right\}}^{l}\;⊕\;W_{d_{5},\left\{1,2,3,6\right\}}^{l}$	user 4: $W_{d_{3},\left\{2,4,5,6\right\}}^{l}\;⊕\;W_{d_{6},\left\{1,2,3,4\right\}}^{l}$
{1,2,3}	{4,5,6}
user 3: $W_{d_{1},\left\{2,3,4,5\right\}}^{2}\;⊕\;W_{d_{2},\left\{1,3,4,5\right\}}^{2}$	user 4: $W_{d_{5},\left\{2,3,4,6\right\}}^{2}\;⊕\;W_{d_{6},\left\{2,3,4,5\right\}}^{2}$
user 3: $W_{d_{1},\left\{2,3,4,6\right\}}^{2}\;⊕\;W_{d_{2},\left\{1,3,4,6\right\}}^{2}$	user 4: $W_{d_{5},\left\{1,2,4,6\right\}}^{2}\;⊕\;W_{d_{6},\left\{1,2,4,5\right\}}^{2}$
user 2: $W_{d_{1},\left\{2,3,5,6\right\}}^{2}\;⊕\;W_{d_{3},\left\{1,2,4,5\right\}}^{2}$	user 5: $W_{d_{4},\left\{1,3,5,6\right\}}^{2}\;⊕\;W_{d_{6},\left\{1,3,4,5\right\}}^{2}$
user 1: $W_{d_{3},\left\{1,2,4,6\right\}}^{2}\;⊕\;W_{d_{2},\left\{1,3,5,6\right\}}^{2}$	user 6: $W_{d_{4},\left\{1,2,5,6\right\}}^{2}\;⊕\;W_{d_{5},\left\{1,3,4,6\right\}}^{2}$
user 1: $W_{d_{3},\left\{1,2,5,6\right\}}^{2}\;⊕\;W_{d_{2},\left\{1,3,4,5\right\}}^{1}$	user 6: $W_{d_{5},\left\{1,2,4,6\right\}}^{1}\;⊕\;W_{d_{4},\left\{2,3,5,6\right\}}^{2}$

**Table 2:** Parallel user delivery when K=7, s=4, $G_{1}^{r}=4$ and $G_{2}^{r}=3$, r∈[35].

{1,2,3,4}	{5,6,7}
user1:$W_{d_{2},\left\{1,3,4\right\}}^{u_{1},1}$⊕$W_{d_{3},\left\{1,2,4\right\}}^{u_{1},1}$⊕$W_{d_{4},\left\{1,2,3\right\}}^{u_{1},1}$	$\mathrm{user}\;5:\underset{x\in \{1,2,3,4\}}{\cup }$$W_{d_{6},\left\{5,7,x\right\}}^{u_{2},1}$⊕$W_{d_{7},\left\{5,6,x\right\}}^{u_{2},1}$
user2:$W_{d_{1},\left\{2,3,4\right\}}^{u_{1},1}$⊕$W_{d_{3},\left\{1,2,4\right\}}^{u_{1},2}$⊕$W_{d_{4},\left\{1,2,3\right\}}^{u_{1},2}$	$\mathrm{user}\;6:\underset{x\in \{1,2,3,4\}}{\cup }$$W_{d_{5},\left\{6,7,x\right\}}^{u_{2},1}$⊕$W_{d_{7},\left\{5,6,x\right\}}^{u_{2},2}$
user3:$W_{d_{2},\left\{1,3,4\right\}}^{u_{1},2}$⊕$W_{d_{1},\left\{2,3,4\right\}}^{u_{1},2}$⊕$W_{d_{4},\left\{1,2,3\right\}}^{u_{1},3}$	$\mathrm{user}\;7:\underset{x\in \{1,2,3,4\}}{\cup }$$W_{d_{6},\left\{5,7,x\right\}}^{u_{2},2}$⊕$W_{d_{5},\left\{6,7,x\right\}}^{u_{2},2}$
user4:$W_{d_{2},\left\{1,3,4\right\}}^{u_{1},3}$⊕$W_{d_{3},\left\{1,2,4\right\}}^{u_{1},3}$⊕$W_{d_{1},\left\{2,3,4\right\}}^{u_{1},3}$	
{1,2,3,5}	{4,6,7}
user1:$W_{d_{2},\left\{1,3,5\right\}}^{u_{1},1}$⊕$W_{d_{3},\left\{1,2,5\right\}}^{u_{1},1}$⊕$W_{d_{5},\left\{1,2,3\right\}}^{u_{1},1}$	$\mathrm{user}\;4:\underset{x\in \{1,2,3,5\}}{\cup }$$W_{d_{6},\left\{4,7,x\right\}}^{u_{2},y_{\left(..\right)}}$⊕$W_{d_{7},\left\{4,6,x\right\}}^{u_{2},y_{\left(..\right)}}$
user2:$W_{d_{1},\left\{2,3,5\right\}}^{u_{1},1}$⊕$W_{d_{3},\left\{1,2,5\right\}}^{u_{1},2}$⊕$W_{d_{5},\left\{1,2,3\right\}}^{u_{1},2}$	$\mathrm{user}\;6:\underset{x\in \{1,2,3,5\}}{\cup }$$W_{d_{4},\left\{6,7,x\right\}}^{u_{2},1}$⊕$W_{d_{7},\left\{4,6,x\right\}}^{u_{2},y_{\left(..\right)}}$
user3:$W_{d_{2},\left\{1,3,5\right\}}^{u_{1},2}$⊕$W_{d_{1},\left\{2,3,5\right\}}^{u_{1},2}$⊕$W_{d_{5},\left\{1,2,3\right\}}^{u_{1},3}$	$\mathrm{user}\;7:\underset{x\in \{1,2,3,5\}}{\cup }$$W_{d_{6},\left\{4,7,x\right\}}^{u_{2},y_{\left(..\right)}}$⊕$W_{d_{4},\left\{6,7,x\right\}}^{u_{2},2}$
user5:$W_{d_{2},\left\{1,3,5\right\}}^{u_{1},3}$⊕$W_{d_{3},\left\{125\right\}}^{u_{1},3}$⊕$W_{d_{1},\left\{235\right\}}^{u_{1},3}$	
{1,2,3,6}	{4,5,7}
user1:$W_{d_{2},\left\{1,3,6\right\}}^{u_{1},1}$⊕$W_{d_{3},\left\{1,2,6\right\}}^{u_{1},1}$⊕$W_{d_{6},\left\{1,2,3\right\}}^{u_{1},1}$	$\mathrm{user}\;4:\underset{x\in \{1,2,3,6\}}{\cup }$$W_{d_{5},\left\{4,7,x\right\}}^{u_{2},y_{\left(..\right)}}$⊕$W_{d_{7},\left\{4,5,x\right\}}^{u_{2},y_{\left(..\right)}}$
user2:$W_{d_{1},\left\{2,3,6\right\}}^{u_{1},1}$⊕$W_{d_{3},\left\{1,2,6\right\}}^{u_{1},2}$⊕$W_{d_{6},\left\{1,2,3\right\}}^{u_{1},2}$	$\mathrm{user}\;5:\underset{x\in \{1,2,3,6\}}{\cup }$$W_{d_{4},\left\{5,7,x\right\}}^{u_{2},1}$⊕$W_{d_{7},\left\{4,5,x\right\}}^{u_{2},y_{\left(..\right)}}$
user3:$W_{d_{2},\left\{1,3,6\right\}}^{u_{1},2}$⊕$W_{d_{1},\left\{2,3,6\right\}}^{u_{1},2}$⊕$W_{d_{6},\left\{1,2,3\right\}}^{u_{1},3}$	$\mathrm{user}\;7:\underset{x\in \{1,2,3,6\}}{\cup }$$W_{d_{5},\left\{4,7,x\right\}}^{u_{2},y_{\left(..\right)}}$⊕$W_{d_{4},\left\{5,7,x\right\}}^{u_{2},2}$
user6:$W_{d_{2},\left\{1,3,6\right\}}^{u_{1},3}$⊕$W_{d_{3},\left\{1,2,6\right\}}^{u_{1},3}$⊕$W_{d_{1},\left\{2,3,6\right\}}^{u_{1},3}$	
⋯ ⋯⋯	⋯ ⋯⋯

There should be 35 partitions in total while the table only shows three partitions.

## Appendix A. Proof of the Converse

Let $T_{1}^{*}$ and $T_{2}^{*}$ denote the optimal rate sent by the server and each user. We first consider an enhance system where every user is served by an exclusive server and user, which both store full files in the database, then we are easy to obtain the following lower bound:

(A1) $$\begin{array}{c} T^{*}\geq \frac{1}{2}\left(1-\frac{M}{N}\right). \end{array}$$

Another lower bound follows similar idea to [1]. However, due to the flexibility of D2D network, the connection and partitioning status between users can change during the delivery phase, prohibiting the direct application of the proof in [1] into the hybrid network considered in this paper. Moreover, the parallel transmission of the server and many users creates abundant different signals in the networks, making the scenario more sophisticated.

Consider the first *s* users with cache contents $Z_{1},...,Z_{s}$. Define $X_{1,0}$ as the signal sent by the server, and $X_{1,1},\ldots ,X_{1,\alpha_{\max}}$ as the signals sent by the $\alpha_{\max}$ users, respectively, where $X_{j,i}\in \left[\left\lfloor 2^{T_{2}^{*}F}\right\rfloor \right]$ for j∈[s] and $i\in [\alpha_{\max}]$. Assume that $W_{1},\ldots ,W_{s}$ are determined by $X_{1,0}$, $X_{1,1},\ldots ,X_{1,\alpha_{\max}}$ and $Z_{1},\ldots ,Z_{s}$. Additionally, define $X_{2,0}$, $X_{2,1},\ldots ,X_{2,\alpha_{\max}}$ as the signals which enable the users to decode $W_{s+1},...,W_{2s}$. Continue the same process such that $X_{\lfloor N/s\rfloor ,0}$, $X_{\lfloor N/s\rfloor ,1},\ldots ,X_{\left\lfloor N/s\right\rfloor ,\alpha_{\max}}$ are the signals which enable the users to decode $W_{s\lfloor N/s\rfloor -s+1},...,W_{s\lfloor N/s\rfloor }$. We then have $Z_{1},\ldots ,Z_{s}$, $X_{1,0},\ldots ,X_{\lfloor N/s\rfloor ,0}$, and

$$X_{1,1},\ldots ,X_{1,\alpha_{\max}},\ldots ,X_{\lfloor N/s\rfloor ,1},\ldots ,X_{\left\lfloor N/s\right\rfloor ,\alpha_{\max}}$$

to determine $W_{1},\ldots ,W_{s\lfloor N/s\rfloor }$. Let

$$X_{1:\alpha_{\max}}≜\left(X_{1,1},\ldots ,X_{1,\alpha_{\max}},\ldots ,X_{\lfloor N/s\rfloor ,1},\ldots ,X_{\left\lfloor N/s\right\rfloor ,\alpha_{\max}}\right).$$

By the definitions of $T_{1}^{*}$, $T_{2}^{*}$ and the encoding function (5), we have

(A2) $$\begin{array}{ccc} & & H\left(X_{1,0},\ldots ,X_{\lfloor N/s\rfloor ,0}\right)\leq \left\lfloor N/s\right\rfloor T_{1}^{*}F, \end{array}$$

(A3) $$\begin{array}{ccc} & & H\left(X_{1:\alpha_{\max}}\right)\leq \left\lfloor N/s\right\rfloor \alpha_{\max}T_{2}^{*}F, \end{array}$$

(A4) $$\begin{array}{ccc} & & H(X_{1:\alpha_{\max}},Z_{1},\ldots ,Z_{s})\leq KMF. \end{array}$$

Consider the cut separating $X_{1,0},\ldots ,X_{\lfloor N/s\rfloor ,0}$, $X_{1:\alpha_{\max}}$, and $Z_{1},\ldots ,Z_{s}$ from the corresponding *s* users. By the cut-set bound and (A2), we have

(A5) $$\begin{array}{cc} \lfloor \frac{N}{s}\rfloor sF & \leq \left\lfloor \frac{N}{s}\right\rfloor T_{1}^{*}F+KMF, \end{array}$$

(A6) $$\begin{array}{cc} \lfloor \frac{N}{s}\rfloor sF & \leq \left\lfloor \frac{N}{s}\right\rfloor T_{1}^{*}F+sMF+\left\lfloor \frac{N}{s}\right\rfloor \alpha_{\max}T_{2}^{*}F. \end{array}$$

Since we have $T^{*}\geq T_{1}^{*}$ and $T^{*}\geq \max\left\{T_{1}^{*},T_{2}^{*}\right\}$ from the above definition, we obtain

(A7) $$\begin{array}{cc} T^{*} & \geq \max_{s\in [K]}\left(s-\frac{KM}{\left\lfloor N/s\right\rfloor }\right), \end{array}$$

(A8) $$\begin{array}{cc} T^{*} & \geq \max_{s\in [K]}\left(s-\frac{sM}{\left\lfloor N/s\right\rfloor }\right)\frac{1}{1+\alpha_{\max}}. \end{array}$$

## Appendix B

We prove that $T_{\mathrm{central}}$ is within a constant multiplicative gap of the minimum transmission delay $T^{*}$ for all values of *M*. To prove the result, we compare them in the following regimes.

- If 0.6393<t<⌊K/α⌋−1, from Theorem 1, we have
  (A9) $$\begin{array}{cc} T^{*} & \geq \left(s-\frac{Ms}{\left\lfloor N/s\right\rfloor }\right)\frac{1}{1+\alpha_{\max}}\\ \; & \overset{\left(a\right)}{\geq }\frac{1}{12}\cdot K\left(1-\frac{M}{N}\right)\frac{1}{1+t}\cdot \frac{1}{1+\alpha_{\max}}, \end{array}$$
  where (a) follows from [1] [Theorem 3]. Then we have
  (A10) $$\begin{array}{cc} \frac{T_{\mathrm{central}}}{T^{*}}\; & \leq 12\cdot \frac{\left(1+\alpha_{\max}\right)\left(1+t\right)}{1+t+\alpha t}\\ \; & =12\cdot \frac{\left(1+\alpha_{\max}\right)}{1+\alpha t/(1+t)}\\ \; & \leq 12\cdot \frac{\left(1+\alpha_{\max}\right)}{1+\alpha \cdot 0.6393/(1+0.6393)}\\ \; & \leq 31, \end{array}$$
  where the last inequality holds by setting $\alpha =\alpha_{\max}$.
- If t>⌊K/α⌋−1, we have
  (A11) $$\begin{array}{cc} \frac{T_{\mathrm{central}}}{T^{*}} & \leq \frac{K\left(1-\frac{M}{N}\right)\frac{1}{1+t+\alpha (\lfloor K/\alpha \rfloor -1)}}{\frac{1}{2}\left(1-\frac{M}{N}\right)}\\ \; & =\frac{2K}{1+t+\alpha (\lfloor K/\alpha \rfloor -1)}\\ \; & \overset{\left(a\right)}{\leq }\frac{2K}{K+KM/N}\\ \; & \leq 2, \end{array}$$
  where (a) holds by choosing α=1.
- If t≤0.6393, setting s=0.275N, we have
  (A12) $$\begin{array}{cc} T^{*} & \geq s-\frac{KM}{\left\lfloor N/s\right\rfloor }\\ \; & \overset{\left(a\right)}{\geq }s-\frac{KM}{N/s-1}\\ \; & =0.275N-t\cdot 0.3793N\\ \; & \geq 0.0325N>\frac{1}{31}\cdot N, \end{array}$$
  where (a) holds since ⌊x⌋≥x−1 for any x≥1. Please note that for all values of *M*, the transmission delay
  (A13) $$T_{\mathrm{central}}\leq \min\left\{K,N\right\}.$$
  Combining with (A12) and (A13), we have $\frac{T_{\mathrm{central}}}{T^{*}}\leq 31.$

## Appendix C

### Appendix C.1. Case α_max_ = $\left\lfloor \frac{K}{2}\right\rfloor$

When $\alpha_{\max}=\left\lfloor \frac{K}{2}\right\rfloor$, we have

(A14) Ru=Ru-f≜∑s=2KKK−1s−1f(K,s)ps−1qK−s+1,

where $R_{\text{u-f}}$ denotes the user’s transmission rate for a flexible D2D network with $\alpha_{\max}=\left\lfloor \frac{K}{2}\right\rfloor$. In the flexible D2D network, at most $\left\lfloor \frac{K}{2}\right\rfloor$ users are allowed to transmit messages simultaneously, in which the user transmission turns to unicast.

Please note that in each term of the summation:

(A15) KK−1s−1f(K,s)≤KK−1s−1K−1−Ks=KK−1+KK−12s−KK−1·K−1s−1≤KK−1s−1K−1+2KKs(K−1)(K−2),

where the last inequality holds by $s\geq \frac{K}{K-1}+\frac{K-2}{K-1}=2$ and

KK−12s−KK−1K−1s−1=K2K−1s−1(K−1)(K−2)·K−2K−1s−KK−1≤K2K−1s−1(K−1)(K−2)·K−2K−1+KK−1s−KK−1+KK−1=2K(K−1)(K−2)·Ks.

Therefore, by (A15), $R_{\text{u-f}}$ can be rewritten as

Ru-f≤KK−1∑s=2KK−1s−1ps−1qK−s+1+2K(K−1)(K−2)∑s=2KKsps−1qK−s+1=KqK−1·∑i=1K−1K−1ipiqK−1−i+2Kq/p(K−1)(K−2)·∑s=2KKspsqK−s=KqK−11−qK−1+2Kq/p(K−1)(K−2)·1−qK−KpqK−1.

### Appendix C.2. Case α_max_ =1

When $\alpha_{\max}=1$, the cooperation network degenerates into a shared link where only one user acts as the server and broadcasts messages to the remaining K−1 users. A similar derivation is given in [35]. In this case, $R_{u}$ can be rewritten as

Ru=∑s=2KsKss−1ps−1qK−s+1≤∑s=2K1+3s+1Ksps−1qK−s+1=∑s=2KKsps−1qK−s+1+3K+1∑s=2KK+1s+1ps−1qK−s+1=qp1−qK−KpqK−1+3q/p2K+11−qK+1−K+1pqK−K(K+1)2p2qK−1=qp1−52KpqK−1−4qK+3(1−qK+1)(K+1)p,

where the inequality holds by the fact that s≥2.

## Appendix D

### Appendix D.1. When α_max_ = $\left\lfloor \frac{K}{2}\right\rfloor$

Recall that $p_{\mathrm{th}}≜1-{\left(\frac{1}{K+1}\right)}^{\frac{1}{K-1}}$, which tends to zero as *K* goes to infinity. We first introduce the following three lemmas.

**Lemma** **A1.**
*Given arbitrary convex function $g_{1}\left(p\right)$ and arbitrary concave function $g_{2}\left(p\right)$, if they intersect at two points with $p_{1}<p_{2}$, then $g_{1}\left(p\right)\leq g_{2}\left(p\right)$ for all $p\in [p_{1},p_{2}]$.*

We omit the proof of Lemma A1 as it is straightforward.

**Lemma** **A2.**
*For memory size 0≤p≤1 and $\alpha_{\max}=\left\lfloor \frac{K}{2}\right\rfloor$, we have*

$$R_{u}\geq R_{\emptyset },\;T_{\mathrm{decentral}}=\frac{R_{s}R_{u}}{R_{s}+R_{u}-R_{\emptyset }},\;\mathrm{for}\;\mathrm{all}\;p\in \left[p_{\mathrm{th}},1\right].$$

**Proof.** When $\alpha_{\max}=\left\lfloor \frac{K}{2}\right\rfloor$, from Equation (20), we have

(A16) Ru=∑s=2KKK−1s−1f(K,s)ps−1(1−p)K−s+1≥KK∑x=1K−1K−1xpx(1−p)K−x=(1−p)1−(1−p)K−1,

where the first inequality holds by letting x=s−1 and $\frac{K}{K-1-\lfloor \frac{K}{s}\rfloor }>\frac{K}{K-1}$. It is easy to show that $\left(1-p\right)\left(1-{\left(1-p\right)}^{K-1}\right)$ is a concave function of *p* by verifying $\frac{\partial^{2}\left(1-p\right)\left(1-{\left(1-p\right)}^{K-1}\right)}{\partial p^{2}}\leq 0$. □

On the other hand, one can easily show that

$$R_{\emptyset }=K{\left(1-p\right)}^{K}$$

is a convex function of *p* by showing $\frac{\partial^{2}R_{\emptyset }\left(p\right)}{\partial p^{2}}\geq 0$. Since the two functions $\left(1-p\right)\left(1-{\left(1-p\right)}^{K-1}\right)$ and $R_{\emptyset }$ intersect at $p_{\mathrm{th}}=1-{\left(\frac{1}{K+1}\right)}^{\frac{1}{K-1}}$ and $p_{2}=1$ with $p_{\mathrm{th}}\leq p_{2}$, from Lemma A1 and (A16), we have

$$R_{u}\geq \left(1-p\right)\left(1-{\left(1-p\right)}^{K-1}\right)\geq R_{\emptyset },$$

for all $p\in [p_{\mathrm{th}},1]$. From Remark 4, we know that $T_{\mathrm{decentral}}=\frac{R_{s}R_{u}}{R_{s}+R_{u}-R_{\emptyset }}$ if $R_{u}\geq R_{\emptyset }$

**Lemma** **A3.**
*For memory size 0≤p≤1 and $\alpha_{\max}=\left\lfloor \frac{K}{2}\right\rfloor$, we have*

$$\frac{R_{s}R_{u}}{R_{s}+R_{u}-R_{\emptyset }}\leq 6T^{*}.$$

**Proof** From (25) and (19), we have

(A17) $$\begin{array}{cc} R_{u} & \leq \frac{K}{K-1}\cdot \left(q-q^{K}\right)+\frac{2K}{\left(K-1)(K-2\right)}\cdot \frac{q}{p}\left(1-q^{K}-Kpq^{K-1}\right)\\ \; & \overset{\left(a\right)}{\leq }\frac{K}{K-1}\cdot \left(q-q^{K}\right)+\frac{2K}{\left(K-1)(K-2\right)}\cdot \frac{q}{p}\left(1-\left(1-Kp\right)-Kpq^{K-1}\right)\\ \; & =\frac{K(3K-2)}{\left(K-1)(K-2\right)}\cdot \left(q-q^{K}\right), \end{array}$$

(A18) $$\begin{array}{cc} R_{s} & =\frac{q}{p}\left(1-q^{K}\right)\overset{\left(b\right)}{\leq }\frac{q}{p}\left(1-\left(1-Kp\right)\right)=Kq, \end{array}$$

where (a) and (b) both follow from inequality

(A19) $$\begin{array}{c} {\left(1-p\right)}^{K}\geq \left(1-Kp\right). \end{array}$$

□

Then, by Remark 4 and (A17), (A18) and definition of $R_{\emptyset }$ in (18), if $\alpha_{\max}=\left\lfloor \frac{K}{2}\right\rfloor$, then

(A20) $$\begin{array}{cc} \frac{R_{s}R_{u}}{R_{s}+R_{u}-R_{\emptyset }} & \leq \frac{Kq\cdot \frac{K(3K-2)}{\left(K-1)(K-2\right)}\left(q-q^{K}\right)}{Kq+\frac{K(3K-2)}{\left(K-1)(K-2\right)}\left(q-q^{K}\right)-Kq^{K}}\\ \; & =\left(3-\frac{2}{K}\right)\cdot q. \end{array}$$

From Theorem 1, we have $T^{*}\geq \frac{1}{2}q$. Thus, we obtain

$$\frac{R_{s}R_{u}}{R_{s}+R_{u}-R_{\emptyset }}\cdot \frac{1}{T^{*}}\leq \frac{\left(3-2/K\right)\cdot q}{q/2}\leq 6-\frac{4}{K}<6.$$

Next, we use Lemmas A2 and A3 to prove that when $\alpha_{\max}=\left\lfloor \frac{K}{2}\right\rfloor$,

$$\begin{array}{c} \frac{T_{\mathrm{decentral}}}{T^{*}}\leq \left\{\begin{array}{ccc} & \max\left\{6,2K{\left(\frac{2K}{2K+1}\right)}^{K-1}\right\}, & p<p_{\mathrm{th}},\;\\ & 6, & p\geq p_{\mathrm{th}}.\; \end{array}\right. \end{array}$$

#### Appendix D.1.1. Case α_max_ = $\left\lfloor \frac{K}{2}\right\rfloor$ and *p* ≥ *p_th_*

In this case, from Lemma A2, we have

$$T_{\mathrm{decentral}}=\frac{R_{s}R_{u}}{R_{s}+R_{u}-R_{\emptyset }}.$$

Thus, from Lemma A3,

$$T_{\mathrm{decentral}}=\frac{R_{s}R_{u}}{R_{s}+R_{u}-R_{\emptyset }}\leq 6T^{*}.$$

#### Appendix D.1.2. Case α_max_ = $\left\lfloor \frac{K}{2}\right\rfloor$ and *p* ≥ *p_th_*

From the definition of $T_{\mathrm{decentral}}$ in (17), we have

(A21) $$\begin{array}{cc} \frac{T_{\mathrm{decentral}}}{T^{*}}\; & =\max\{\frac{R_{\emptyset }}{T^{*}},\frac{R_{s}R_{u}}{R_{s}+R_{u}-R_{\emptyset }}\cdot \frac{1}{T^{*}}\}. \end{array}$$

From Lemma A3, we know that

(A22) $$\begin{array}{c} \frac{R_{s}R_{u}}{R_{s}+R_{u}-R_{\emptyset }}\cdot \frac{1}{T^{*}}\leq 6, \end{array}$$

and thus only focus on the upper bound of $R_{\emptyset }/T^{*}$.

According to Theorem 1, $T^{*}$ has the following two lower bounds: $T^{*}\geq \frac{1-p}{2}$, and

$$\begin{array}{c} T^{*}\geq \max_{s\in [K]}\left(s-\frac{KM}{\left\lfloor N/s\right\rfloor }\right)\geq \max_{s\in [K]}\left(s-\frac{KM}{N/(2s)}\right). \end{array}$$

Let $R_{1}^{*}\left(p\right)≜\frac{1}{2}\left(1-p\right)$ and $R_{2}^{*}\left(p\right)≜\left(K-2K^{2}p\right)$, then we have

$$T^{*}\geq \max\left\{R_{1}^{*}\left(p\right),R_{2}^{*}\left(p\right)\right\}.$$

Here $R_{\emptyset }/R_{1}^{*}\left(p\right)$ and $R_{\emptyset }/R_{2}^{*}\left(p\right)$ both are monotonic functions of *p* according to the following properties:

$$\begin{array}{cc} \frac{\partial \left(R_{\emptyset }/R_{1}^{*}\left(p\right)\right)}{\partial p} & =\frac{\partial \left(2K{\left(1-p\right)}^{K-1}\right)}{\partial p}\leq 0,\\ \frac{\partial \left(R_{\emptyset }/R_{2}^{*}\left(p\right)\right)}{\partial p} & =\frac{\partial \left(q^{K}/\left(1-2Kp\right)\right)}{\partial p}\\ \; & =\frac{Kq^{K-1}\left(1+2(K-1)p\right)}{{\left(1-2Kp\right)}^{2}}\geq 0. \end{array}$$

Additionally, notice that if p=0, then $\frac{R_{\emptyset }}{R_{2}^{*}\left(p\right)}=1<\frac{R_{\emptyset }}{R_{1}^{*}\left(p\right)}$, and if p=1, $\frac{R_{\emptyset }}{R_{2}^{*}\left(p\right)}>\frac{R_{\emptyset }}{R_{1}^{*}\left(p\right)}=1$. Therefore, the maximum value of $R_{\emptyset }/\max\left\{R_{1}^{*},R_{2}^{*}\right\}$ is chosen at $p=\frac{1}{2K+1}$ which satisfying $R_{1}^{*}\left(\frac{1}{2K+1}\right)=R_{2}^{*}\left(\frac{1}{2K+1}\right)$, implying that

(A23) $$\begin{array}{c} \frac{R_{\emptyset }}{T^{*}}\leq \frac{R_{\emptyset }\left(\frac{1}{2K+1}\right)}{R_{1}^{*}\left(\frac{1}{2K+1}\right)}=2K{\left(\frac{2K}{2K+1}\right)}^{K-1}. \end{array}$$

From (A21)–(A23), we obtain the following equality:

$$\frac{T_{\mathrm{decentral}}}{T^{*}}\leq \max\left\{2K{\left(\frac{2K}{2K\;+\;1}\right)}^{K\;-\;1},6\right\}.$$

### Appendix D.2. When α_max_ = 1

From Equation (24), we obtain that

(A24) $$\begin{array}{cc} R_{u} & \leq \frac{q}{p}\left(1-\frac{5}{2}Kpq^{K-1}-4q^{K}+\frac{3(1-q^{K+1})}{(K+1)p}\right)\\ \; & \leq \frac{q}{p}\left(1-\frac{5}{2}Kpq^{K-1}-4q^{K}+\frac{3(K+1)p}{(K+1)p}\right)\\ \; & =\frac{q}{p}\left(4\cdot \left(1-q^{K}\right)-\frac{5}{2}Kpq^{K-1}\right)\\ \; & \leq \frac{q}{p}\left(4\cdot \left(1-q^{K}\right)\right)\\ \; & =4R_{s}, \end{array}$$

where the second inequality holds by (A19) and the last equality holds by the definition $R_{s}≜\frac{q}{p}\left(1-q^{K}\right)$ in (19). On the other hand, rewrite the second lower bound of $T^{*}$:

(A25) $$\begin{array}{c} T^{*}\geq \max_{s\in [K]}\left(s-\frac{sM}{\left\lfloor N/s\right\rfloor }\right)\frac{1}{1+\alpha_{\max}}. \end{array}$$

From the result in [2] (Appendix B), we have

(A26) $$\begin{array}{c} \frac{R_{s}}{\max_{s\in [K]}\left(s-\frac{sM}{\left\lfloor N/s\right\rfloor }\right)}\leq 12. \end{array}$$

Combining (A24)–(A26), we have

(A27) $$\begin{array}{c} \frac{R_{s}}{T^{*}}\leq 12\left(1+\alpha_{\max}\right),\;\frac{R_{u}}{T^{*}}\leq 48\left(1+\alpha_{\max}\right).\; \end{array}$$

If $p\leq p_{\mathrm{th}}$, by (A27) and since $R_{\emptyset }\leq T_{\mathrm{decentral}}\leq R_{s}$ (see Remark 4), we have

(A28) $$\begin{array}{c} \frac{T_{\mathrm{decentral}}}{T^{*}}\leq \frac{R_{s}}{T^{*}}\leq 12\left(1+\alpha_{\max}\right)=24, \end{array}$$

the last equality holds by the fact $\alpha_{\max}=1$.

If $p\geq p_{\mathrm{th}}$, from Lemma A2, we have $R_{u}\geq R_{\emptyset }$ and

(A29) $$\begin{array}{cc} \frac{T_{\mathrm{decentral}}}{T^{*}} & =\frac{\frac{R_{s}R_{u}}{R_{s}+R_{u}-R_{\emptyset }}}{T^{*}}\\ \; & \leq \frac{\min\{R_{u},R_{s}\}}{T^{*}}\\ \; & \leq \min\{12\left(1+\alpha_{\max}\right),48\left(1+\alpha_{\max}\right)\}\\ \; & =24, \end{array}$$

where the second inequality holds by (A27) and the last equality is from the fact $\alpha_{\max}=1$ in this case.
